# Superprotonic conduction of intrinsically zwitterionic microporous polymers based on easy-to-make squaraine, croconaine and rhodizaine dyes†

**DOI:** 10.1039/d2na00177b

**Published:** 2022-06-08

**Authors:** Dominic Taylor, Xuanhe Hu, Can-Min Wu, John M. Tobin, Zuzana Oriou, Jun He, Zhengtao Xu, Filipe Vilela

**Affiliations:** School of Chemical Engineering and Light Industry, Guangdong University of Technology Guangzhou Guangdong 510006 China junhe@gdut.edu.cn; School of Engineering and Physical Sciences, Heriot-Watt University Edinburgh EH14 4AS UK f.vilela@hw.ac.uk; Materials Innovation Factory and Department of Chemistry, University of Liverpool Crown Street Liverpool L69 7ZD UK; Institute of Materials Research and Engineering 2 Fusionopolis Way, Innovis Building Singapore 138634 zhengtao@imre.a-star.edu.sg

## Abstract

Porous organic polymers (POPs) have been prepared *via* a novel metal free polycondensation between a tritopic indole-based monomer and squaric, croconic and rhodizonic acids. Each of the three POPs exhibited high BET surface areas (331–667 m^2^ g^−1^) and zwitterionic structures. Impedance measurements revealed that the intrinsic POPs were relatively weak proton conductors, with a positive correlation between the density of oxo-groups and the proton conduction. Doping the materials with LiCl vastly improved the proton conductivity up to a value of 0.54 S cm^−1^ at 90 °C and 90% relative humidity.

## Abstract 2

Porous organic polymers (POPs) have been prepared *via* a novel metal-free polycondensation between a tritopic indole-based monomer and squaric, croconic and rhodizonic acids.

## Graphical abstract

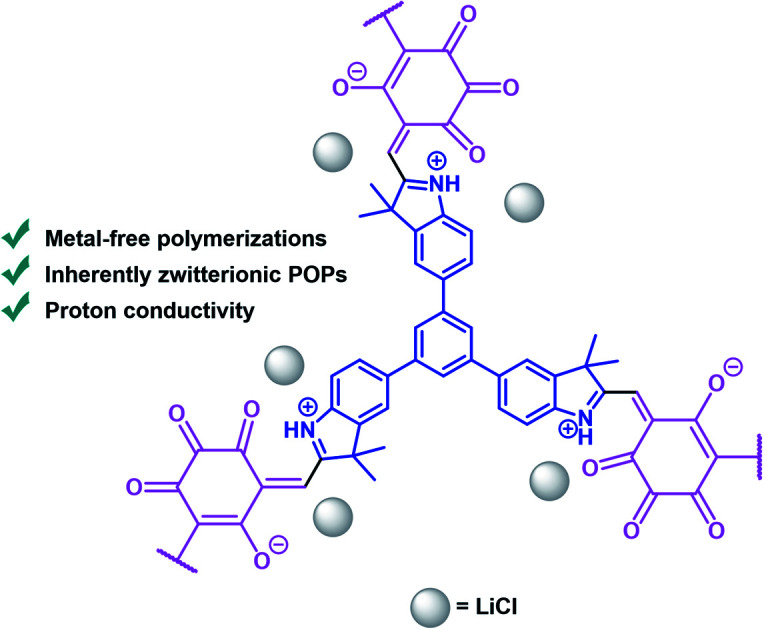

## Introduction

Ionic conduction, vital for energy technologies such as fuel cells and batteries, remains a challenging property to optimize in the solid state, as the mobility of protons and other ions depends on many structural and environmental factors.^1–4^ Of general importance is the number of charge carriers, *e.g.*, in the form of built-in –SO_3_H or other ionic/acidic groups, and of ionic guests and water molecules introduced into the channels of the porous host. These charge carriers should have freedom to undergo dynamic motion and should exhibit relatively weak interactions with the host framework. Furthermore, they should be optimally spaced and orientated for passing along the H^+^, Li^+^ or other mobile ions.^5^ It is therefore a delicate balancing act to modify the structure and functions for achieving higher ionic conductivity.

In this context, metal–organic frameworks (MOF),^6^ and porous organic polymers (POP),^7–9^ offer structural and functional modifiability (largely from the highly variable organic building blocks),^10,11^ and therefore great opportunity to explore and improve solid state ionic conduction properties.^1,3,4^ Incidentally, POP frameworks offer the advantage of stability, especially to the aqueous acidic/basic conditions common in fuel cell setups as compared with many hydrolytically labile MOFs. Equipping the framework backbones with sulfonic and other acidic groups is a tried-and-true method, as dense arrays of appended –SO_3_H groups can help reach record level of proton conductivity.^12–14^ Many other functions have been also tried to improve ionic conductivity, including phosphonic acid,^15^ Troger's base (for anion exchange membranes),^16^ and heterocyclic guests such as 1*H*-imidazole or 1*H*-1,2,3-triazole (*e.g.* for the non-diffusive relay-like proton transport pathway).^17^

On the other hand, doping ionic guests such as LiCl into the host matrix offers a potentially versatile approach to improving ionic conductivity, because of the diverse array of ionic guests that can be post-synthetically inserted for optimization studies.^18–20^ For this, a host material exhibiting strong ionic character in the form of a zwitterionic POP will facilitate uptake of the ionic dopants of inorganic salts. Such salts are hydrophilic and well-hydrated species will be reluctant to penetrate the typically less polar organic matrix of POPs. Zwitterionic POPs remain, however, surprisingly rare. For example, the commonly used amine-carbonyl condensations using 1,3,5-triformylphloroglucinol do not generate polymer frameworks with sufficient ionic character, and extra base/acid groups have to be appended to impart distinct zwitterionic properties.^21–26^ Although zwitterionic POPs have recently started to be explored as ionic conduction materials, studies on inserting ionic salt (*e.g.*, LiCl) dopants for boosting conductivity remains unknown for zwitterionic POP systems.

Reported here is an efficient synthesis of a series of zwitterionic POPs that can be LiCl-doped (by soaking in a saturated THF solution) to achieve proton conductivity as high as 0.54 S cm^−1^ at 90 °C and 90% relative humidity. The distinct zwitterionic character of the polymer framework stems from the condensation between indole and the oxocarbon anions of squaric (SQ), croconic (CR) and rhodozonic (RH) acids. SQ and CR in particular have shown importance as precursors to near-infrared (NIR) absorbing dyes and their ability to undergo condensation reactions with indole, pyrrole or aniline derivatives can generate a plethora of possible structures.^27^ This includes examples of POPs featuring squaraine groups spanning applications such as photocatalysis and energy storage.^28–30^ However, to our knowledge CR and RH have not yet been successfully incorporated into POPs. Our key hypothesis here is that the larger CR and RH, with higher density of the polar oxo-groups, may facilitate the uptake of the ionic guests and enhance proton transport throughout the polymer matrix.

## Results and discussion

### Polymer synthesis and characterization

**Scheme 1:**
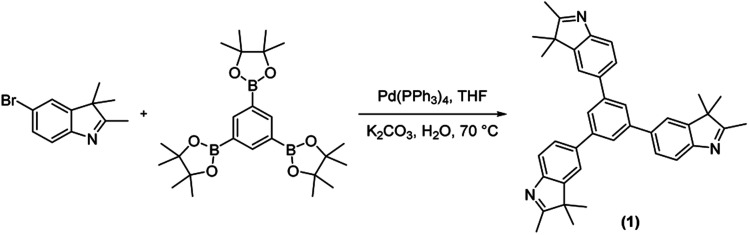
Synthesis of tritopic indole linker through Suzuki–Miyaura cross-coupling.

**Scheme 2:**
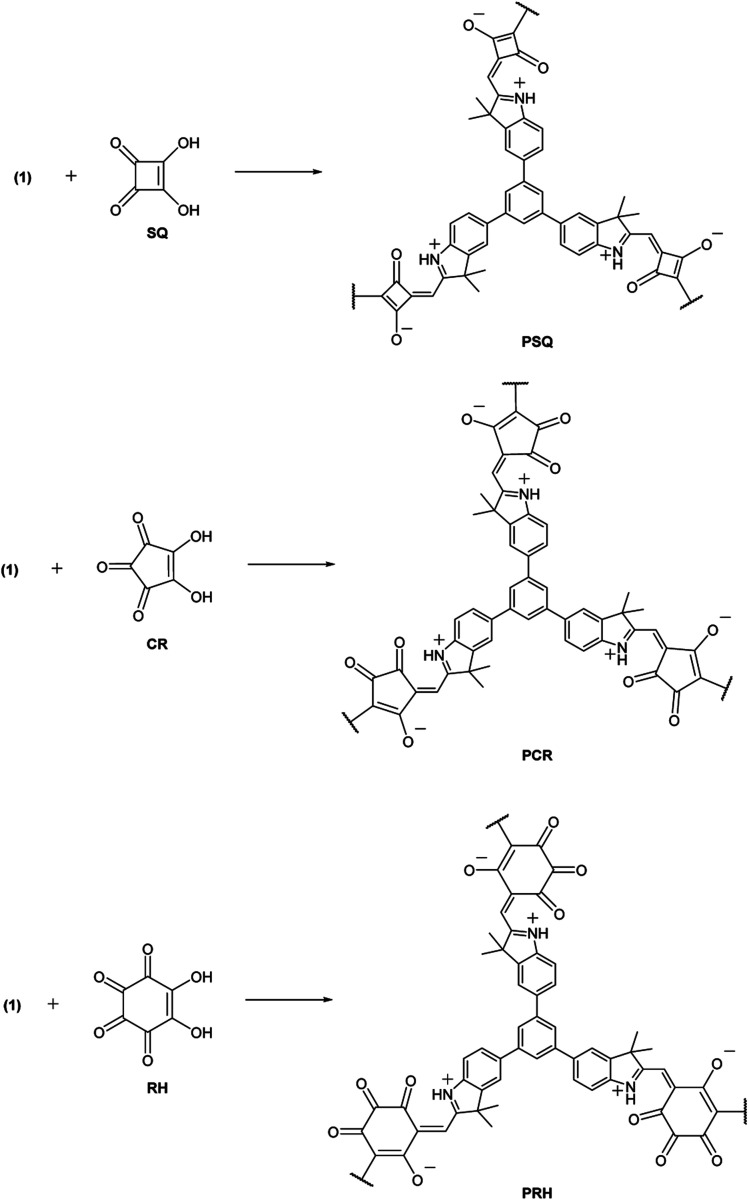
Synthesis of three POPs through polycondensation of 1 with SQ, CR and RH. Reaction conditions: quinoline (6 mol%), butanol/toluene (v/v 1 : 1), 72 h, Dean–Stark apparatus with 3 Å molecular sieves.

For constructing the polymer framework, the tritopic indole-based monomer (1) was prepared *via* the Suzuki–Miyaura cross-coupling between 1,3,5-benzenetriboronic acid pinacol ester and 2,3,3-trimethyl-5-bromo-3*H*-indole (Scheme 1). Subsequent polycondensation of 1 with SQ, CR and RH acids yielded three novel POP materials (Scheme 2). SQ, along with the lesser investigated CR and RH acids, are known to undergo condensation reactions with electron-rich aromatic molecules including pyrrole, indole or aniline derivatives, with water being the condensation by-product.^27^ This approach has been implemented to yield a variety of conjugated poly(squaraines), showing a modulation in the bandgap as well as a bathochromic shift in absorbance values.^31^ Therefore, we employed the electron-rich indole 1 in three metal-free polycondensation reactions utilizing SQ, CR and RH acids to synthesize three POPs (PSQ, PCR and PRH respectively), each with an alternating acid–indole polymeric network. The reactions were performed with quinoline in a butanol/toluene (1 : 1) solvent mixture under a nitrogen atmosphere at 120 °C for 72 h (Scheme 2). After cooling, the precipitate was filtered, washed and purified *via* Soxhlet extraction with toluene, chloroform and methanol then subsequently dried *in vacuo*. The POPs were isolated with excellent recoveries in the range of 85–97%. In short, we were thus able to integrate SQ, CR and RH dyes into a POP structure, without using metal catalysts in the polymerization step.^32–41^

The resulting POPs were characterized *via* spectroscopic methods and other analytical methods were employed to determine their physical characteristics. Analysis of FTIR spectroscopy proved difficult to determine formation of the polymers. However, a sharp band at ∼2960 cm^−1^ in 1 indicates the presence of methyl groups found on the indole (Fig. S2†). This peak, while still present, reduces significantly in the spectra of the POPs, indicating the formation of a bond between the lone methyl group and the acid. Solid state ^13^C NMR analysis was also employed as a further spectroscopic method to determine the functional groups within the POPs. Each group in the PSQ and PCR spectra was well defined. A detailed assignment of the spectra peaks can be found in the ESI (Fig. S4–S6†). However, PRH showed some anomalies when compared to the PSQ and PCR spectra. The most notable difference was the near disappearance of the signals around 180 ppm which are indicative of a ketone functional group that can be found in RH. A new peak at 67 ppm also arises though this can be explained as a shift in the C–OH (O^−^) peak as it falls within the standard range of this functional group.

**Fig. 1:**
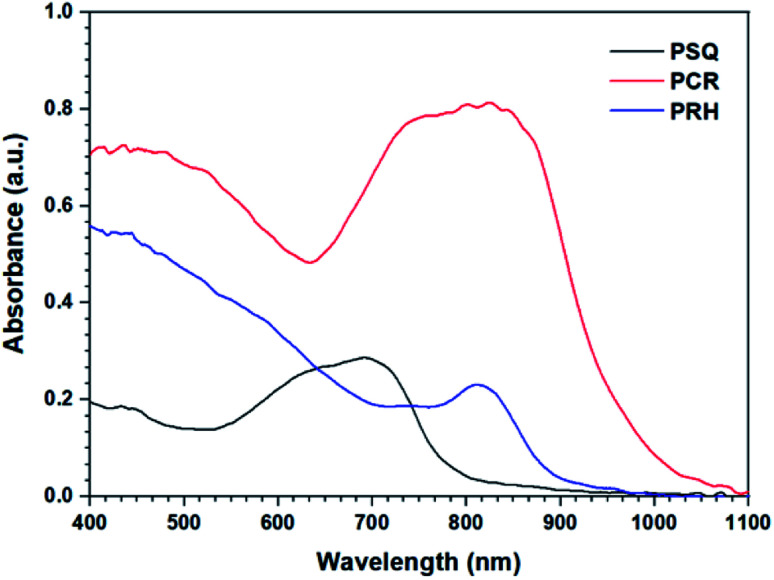
UV-Vis spectra of PSQ, PCR and PRH with local *λ*_max_ ranging from 700–850 nm.

UV-Vis spectroscopy of the POPs (Fig. 1) show a large bathochromic shift into the visible red and near IR region of the spectrum. While PSQ possesses an absorption maxima (*λ*_max_) located at 700 nm, both PCR and PRH have *λ*_max_ located at approximately 800 nm. Interestingly, although PCR and PRH display similar absorbance maxima, the spectra are somewhat dissimilar. PCR exhibits a very broad absorption beginning at 1050 nm and dipping at 630 nm. However, PRH shows only a small local maximum at ∼800 nm which is relatively weak compared to the absorbance between 400-600 nm.

The thermal stability of the POPs was assessed using thermogravimetric analysis (TGA, Fig. S9–S11†) in the temperature region of 25–950 °C under a flow of CO_2_ using an empty alumina crucible as the reference sample. The POPs exhibited a slight change in mass when heating to 100 °C: this could be due to residual solvent trapped in the pores. Decomposition of the POPs began in the region of 150–200 °C and continued until 550 °C, at which point less than 10% of the initial mass was remaining. In comparison to other POPs, those reported here exhibited low thermal stability. This could suggest that the connection formed *via* the polycondensation is susceptible to thermal cleavage.

The surface area of the three POPs was measured *via* N_2_ and CO_2_ gas adsorption experiments at 77 K and 273 K respectively (Table 1, Fig. S12–S23†). The CO_2_ adsorption measurements revealed that the three materials exhibited high BET surface areas (385–472 m^2^ g^−1^) although utilizing N_2_ as the probe molecule led to much lower surface areas (2.3–106 m^2^ g^−1^). This discrepancy most likely stems from the use of N_2_ at cryogenic temperatures, which has been reported to kinetically restrict access to smaller micropores.^42–44^ By comparison, the use of CO_2_ as the probe molecule utilizes higher temperatures and pressures, allowing for faster diffusion and analysis of smaller micropores. For each of the POPs, type 1 CO_2_ adsorption isotherms were observed, which is typical for microporous polymers. The pore size distribution and pore volumes were modelled using the QSDFT (N_2_ isotherms) and the Monte-Carlo models (CO_2_ isotherms), revealing the presence of mainly micropores with additional mesopores.^45^ The pore volumes that were measured by N_2_ gas sorption were particularly low (0.005–0.09 cm^3^ g^−1^), although the range of values obtained through CO_2_ gas sorption experiments (0.131–0.220 cm^3^ g^−1^) were more in line with values expected for POPs. The origin of this could potentially be due to inability of the N_2_ probe gas to fully access the microporous structures of the three POPs, thus underestimating the pore volume significantly. The morphology of PSQ, PCR and PRH were all analyzed *via* scanning electron microscopy (SEM), revealing textured surfaces (Fig. S24–S26†). PCR and PRH both exhibited globular morphologies while PSQ was less well defined in its texture.

**Table 1:** Summary of the surface area and porous features of the synthesised POPs

Material	*S*_BET_/m^2^ g^−1^	Average pore width/nm	Pore volume/cm^3^ g^−1^
PSQ	106^a^	2.382	0.09
	385^b^	0.822	0.220
PCR	2.3^a^	3.096	0.005
	405^b^	0.458	0.131
PRH	42^a^	2.6	0.056
	472^b^	0.785	0.172

a Measured using N_2_ gas at 77 K.

b Measured using CO_2_ gas at 273 K.

### Proton conductivity measurements

**Fig. 2:**
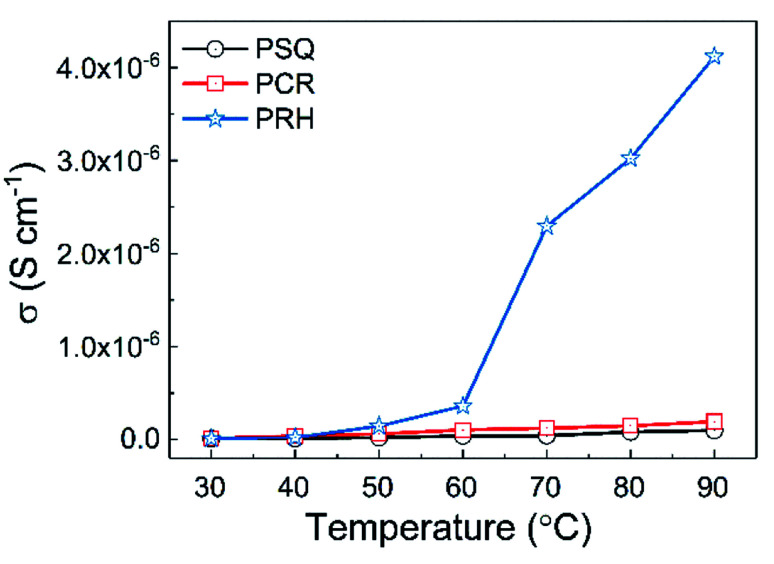
Proton conductivity of PSQ, PCR, PRH at different temperatures (from 30 °C to 90 °C) and 90% relative humidity.

The proton conductivities of pristine POPs were first measured by impedance spectrometry from 30 to 90 °C under 90% relative humidity (Fig. 2, S27 and S28†). It turned out that PSQ, PCR and PRH exhibits relatively low conductivities in the range of 0.98 to 41 × 10^−7^ S cm^−1^ at 90 °C and 90% relative humidity. All of the POPs displayed an increase in conductivity with increasing temperature, which can be attributed to the enhanced diffusivity and ionic mobility at elevated temperature.^46^ Moreover, the proton conductivities at temperatures above 50 °C follow the order PSQ < PCR < PRH, indicating that POPs with a higher density of the polar oxo-groups may facilitate binding of hydrated proton by hydrogen-bonding and thus promote proton transport throughout the polymer matrix.^47–49^

**Fig. 3:**
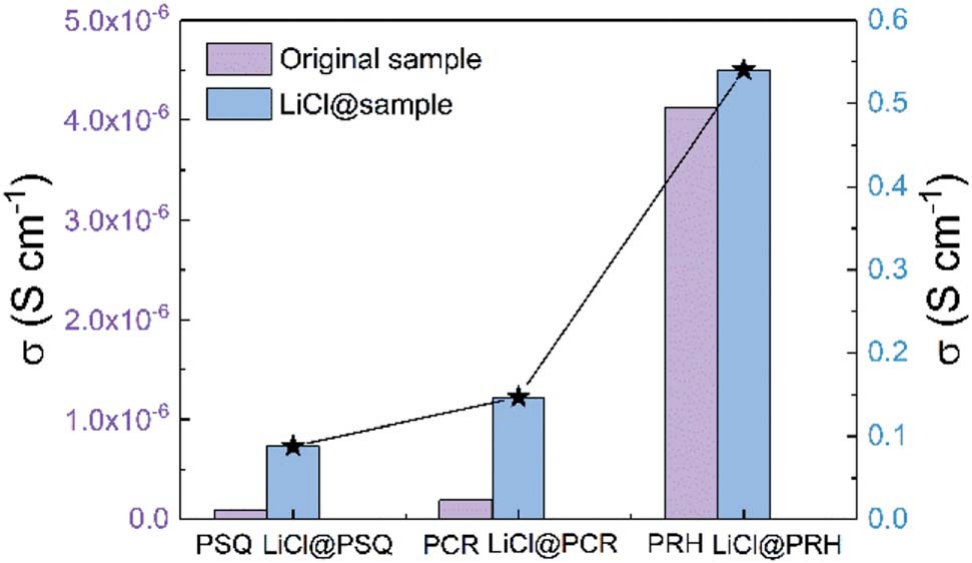
Proton conductivity of PSQ, PCR, PRH and LiCl@PSQ, LiCl@PCR, LiCl@PRH at 90 °C and 90% relative humidity.

Considering that SQ and CR have shown importance as precursors to NIR dyes, the effect of illumination on proton conductivities of POPs was explored. The proton conductivities of the three POPs were then measured after being illuminated by a red LED for different times at 90 °C and 90% relative humidity (Fig. S29 and S30†). However, our results show that illumination has a negligible and negative association with the proton conductivity. As the proton conductivities of the intrinsic POPs were all relatively low, we sought to increase the proton conductivities by doping the material with charge carriers. This is a method that has been previously explored with other proton conductive materials using ionic salts (*e.g.*, LiCl, Cs_2_CO_3_),^18,20^ or electrolytes (*e.g.* imidazole, triazole).^19,50^ With this in mind, we doped PSQ, PCR and PRH with LiCl by soaking the POPs in LiCl/THF for 2 days at room temperature (Fig. S31–S36, see ESI for synthetic details†). This led to six orders of magnitude improvement in the proton conductivity, with values in the range of 0.88 to 5.4 × 10^−1^ S cm^−1^ at 90 °C and 90% relative humidity, which are comparable to the conventional Nafion (0.08 S cm^−1^, Fig. 3, Table S1†).^18^ According to previous studies, this huge increase in the conductivity may have be achieved by facilitating Li^+^ ion movements in the POPs. It is worth noting that after introducing LiCl, the proton conductivities kept the order PSQ < PCR < PRH, further confirming POPs rich in oxo-groups can boost proton transport. As a result, the greatest proton conductivity of 5.4 × 10^−1^ S cm^−1^ for LiCl@PRH was obtained at 90 °C and 90% relative humidity.^51^ In addition, the activation energy (*E*_a_) of the proton conduction in three POPs were estimated from Arrhenius plots to be 0.26–0.40 eV (Fig. S30c–S35c†). Such *E*_a_ values are typical for the Grotthuss mechanism (*E*_a_ < 0.4 eV), which involves proton hopping between adjacent media accompanied by rotational reorientation motion.^52–55^

## Conclusions

In conclusion, a novel method to prepare POPs *via* the metal free polycondensation between a tritopic indole-based linker and squaric, croconic and rhodizonic acids has been developed. Impedance measurements carried out on the pristine polymer materials revealed that they were relatively weak proton conductors. A positive correlation between the proton conductivities at high temperature and the density of oxo-groups in the POPs was observed, suggesting that these groups facilitate proton binding through hydrogen bonding. Doping these materials with LiCl led to vastly improved proton conduction up to 5.4 × 10^−1^ S cm^−1^ at 90 °C and 90% relative humidity.

## Funding sources

This work was partially funded by the National Natural Science Foundation of China (21871061) and Science and Technology Planning Project of Guangdong Province (2021A0505030066). DT and FV acknowledge the EPSRC funded (EP/L016419/1) CRITICAT Centre for Doctoral Training for Dominic Taylor's PhD. ZX acknowledges a GRF grant from the Research Grants Council of HKSAR (CityU 11303519).

## Author contributions

The manuscript was written through contributions of all authors. All authors have given approval to the final version of the manuscript.

## Conflicts of interest

The authors declare no competing financial interest.

## Supplementary Material

NA-004-D2NA00177B-s001
