# Supplementary material for: Superprotonic conduction of intrinsically zwitterionic microporous polymers based on easy-to-make squaraine, croconaine and rhodizaine dyes
Source: Nanoscale Adv. 2022 Jun 8;4(13):2922–8. doi: 10.1039/d2na00177b (PMC9416968; doi:10.1039/d2na00177b)
Supplement: NA-004-D2NA00177B-s001 [file NA-004-D2NA00177B-s001.pdf]

Electronic Supplementary Material (ESI) for Nanoscale Advances.
This journal is © The Royal Society of Chemistry 2022

Superprotonic Conduction of Intrinsically Zwitterionic Microporous Polymers Based on Easy-to-Make Squaraine, Croconaine and Rhodizaine Dyes

Electronic Supplementary Information

Dominic Taylor,[2,⊥] Xuanhe Hu,[1,⊥] Can-Min Wu,[1] John M. Tobin,[2,⊥] Zuzana Oriou,[3] Jun He,[1,*] Zhengtao Xu,[4,*] and Filipe Vilela.[2,*]

[1]School of Chemical Engineering and Light Industry, Guangdong University of Technology, Guangzhou, Guangdong 510006, China.

[2] School of Engineering and Physical Sciences, Heriot-Watt University, Edinburgh, EH14 4AS, United Kingdom.

[3] Materials Innovation Factory and Department of Chemistry, University of Liverpool, Crown Street, Liverpool, L69 7ZD, United Kingdom.

[4] Institute of Materials Research and Engineering, 2 Fusionopolis Way, Innovis Building, Singapore 138634.

E-mail: junhe@gdut.edu.cn, zhengtao@imre.a-star.edu.sg, f.vilela@hw.ac.uk

⊥ These authors contributed equally to this work.

1. Experimental Methods

Synthesis of 1,3,5-tris(2,3,3-trimethyl-3H-indol-5-yl)benzene (1)

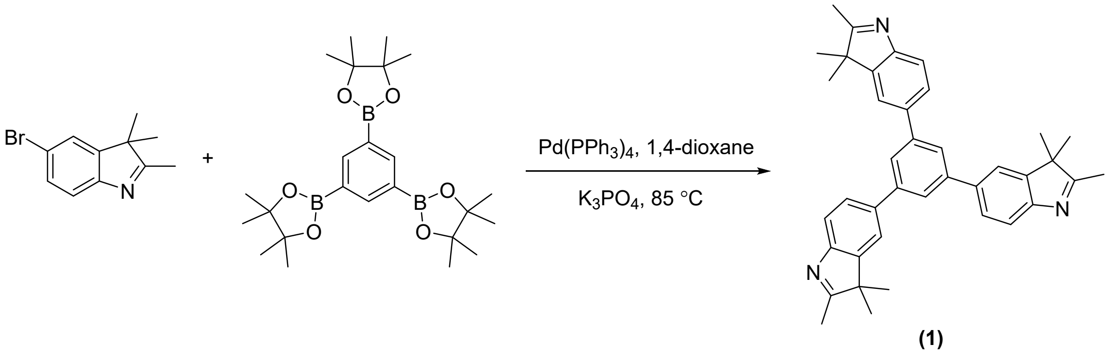

(1)

A two-neck round-bottomed flask (100 mL) was loaded with a magnetic stirring bar and 1,3,5-tris(4,4,5,5-tetramethyl-1,3,2-dioxaborolan-2-yl)benzene (2.0 g, 4.38 mmol), K_3PO_4 (8.4 g, 39.57 mmol) and $Pd(PPh_3)_4$ (250.0 mg, 0.22 mmol), followed by repetition of evacuation and refilling with N_2 thrice. Dry 1,4-dioxane (50 mL) was degassed for 15 minutes and injected into the flask *via* cannula under an N_2 stream. 5-Bromo-2,3,3-trimethyl indole (3.7 g, 15.54 mmol) was added under a N_2 atmosphere. The flask was then connected to a condenser and the mixture was was heated to 85 °C and stirred overnight. The obtained mixture was poured into water (50 mL) and extracted with ethyl acetate (3 × 150 mL). The combined organic layer was then washed with water (3 × 100 mL) and dried with anhydrous Na_2SO_4. Crude product was obtained after the removal of organic solvent using a rotary evaporator. Further purification by column chromatography (silica gel, with EtOAc as the eluent) afforded a pale-yellow solid (1.7 g, 71%). ^{1}H NMR (300 MHz, $CDCl_3$): δ_{ppm} = 1.37 (s, 18 H), 2.32 (s, 9 H), 7.59 (s, 3 H), 7.65 (s+d, 6 H), 7.76 (s, 3 H).

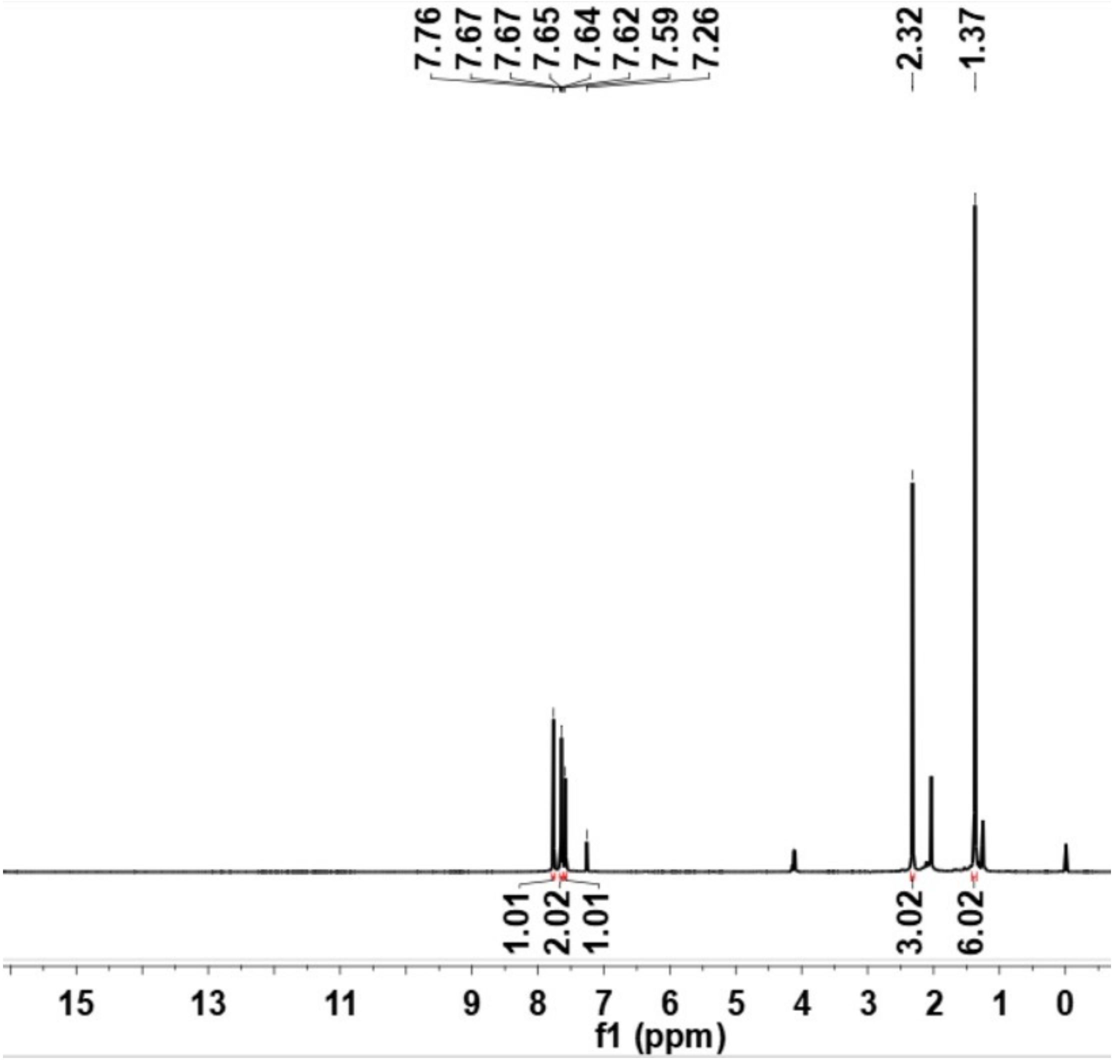

Fig. S1 ^{1}H NMR of compound **1** (300 MHz, 30 °C, $CDCl_3$) δ_{ppm} = 1.37 (s, 6 H), 2.32 (s, 3 H), 7.59 (s, 1 H), 7.65 (s+d, 2 H), 7.76 (s, 1 H).

Synthesis of polysquaraine CPP (PSQ)

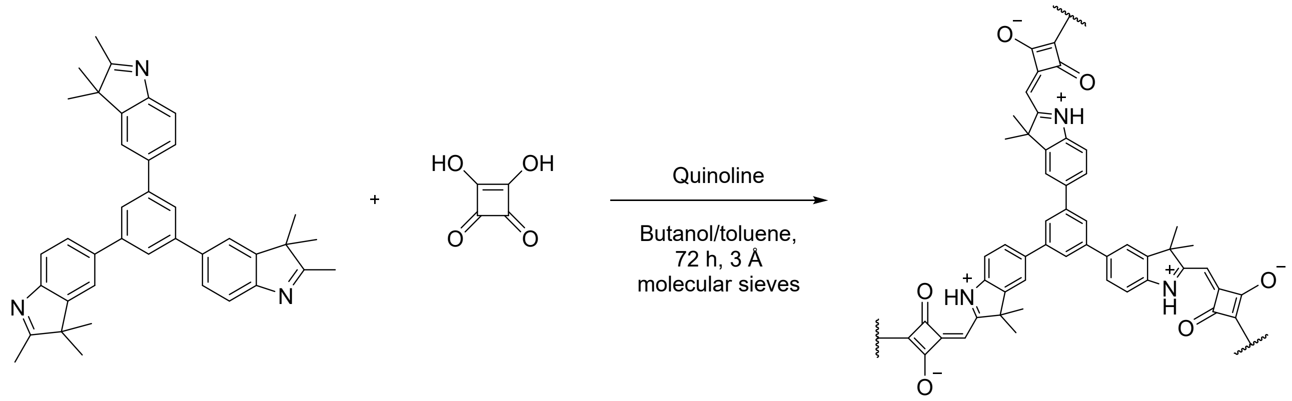

1 **SQ** **PSQ**

1,3,5-Tris(2,3,3-trimethyl-3H-indol-5-yl)benzene (300 mg, 0.545 mmol), squaric acid (94 mg, 0.82 mmol), and quinoline (20 mg, 0.11 mmol) were added to a flask, dissolved in a butanol/toluene mixture (20 mL, v/v 1:1), and deoxygenated *via* bubbling with N_2 for 10 min. The flask was fitted with a Dean-Stark condenser containing molecular sieves (3 Å) and then attached to a condenser. The reaction was heated to 130 °C and left to react over 3 days. After this time, a dark, insoluble solid had formed. The precipitate was filtered and washed with H_2O, acetone and THF. The resulting polymer was purified *via* Soxhlet extraction with toluene (24 h) and chloroform (24 h) and dried *in vacuo* to afford 350 mg (96%) of a metallic red powder.

Synthesis of polycroconaine CPP (PCR)

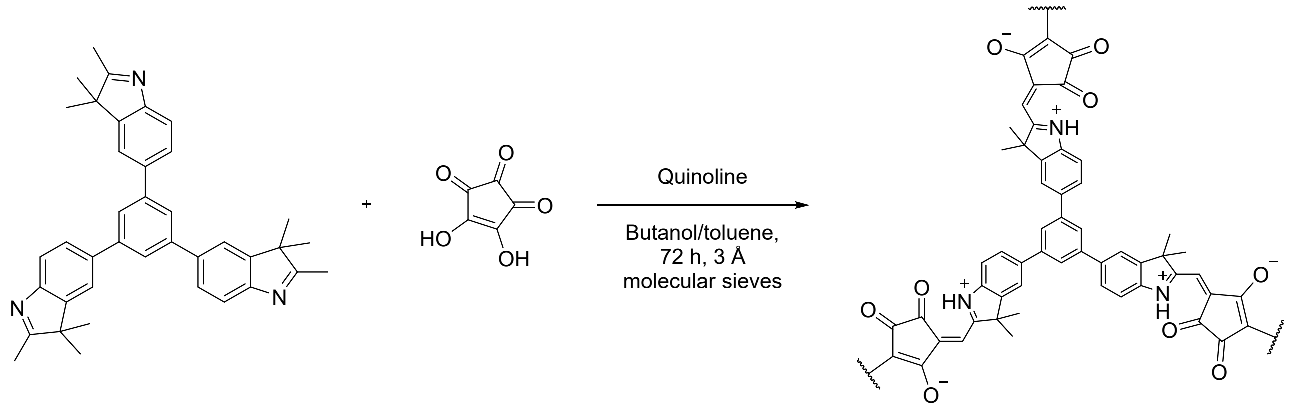

1 CR PCR

1,3,5-Tris(2,3,3-trimethyl-3H-indol-5-yl)benzene (300 mg, 0.545 mmol), croconic acid (116 mg, 0.82 mmol), and quinoline (20 mg, 0.11 mmol) were added to a flask, dissolved in a butanol/toluene mixture (20 ml; v/v 1:1), and deoxygenated *via* bubbling with N_2 for 10 min. The reaction flask was equipped with Dean-Stark condenser containing molecular sieves (3A) and then attached to a condenser. The reaction was heated to 130 °C and left to react over 3 days. After this time, a dark, insoluble solid had formed. The precipitate was filtered and washed with H_2O, acetone and THF. The resulting polymer was purified *via* Soxhlet extraction with toluene (24 h) and chloroform (24 h) and dried *in vacuo* to afford 330 mg (85%) of a metallic green powder.

Synthesis of polyrhodizaine CPP (PRH)

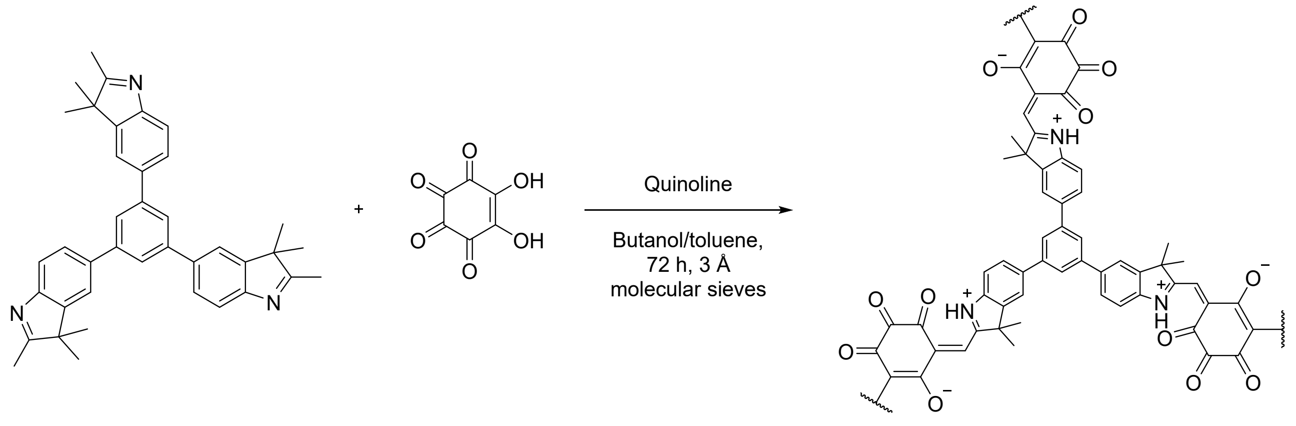

1 **RH** **PRH**

1,3,5-Tris(2,3,3-trimethyl-3H-indol-5-yl)benzene (300 mg, 0.545 mmol), rhodizonic acid (169 mg, 0.2 mmol), and quinolone (16 mg, 0.11 mmol) were added to a flask, dissolved in a butanol/toluene mixture (20 mL; v/v 1:1), and deoxygenated *via* bubbling with N_2 for 10 min. The flask was fitted with a Dean-Stark condenser with molecular sieves (3A) and then attached to a condenser. The reaction was heated to 130 °C and left to react over 3 days. After this time, a dark, insoluble solid had formed. The precipitate was filtered and washed with H_2O, acetone and THF. The resulting polymer was purified *via* Soxhlet extraction with methanol (24 h) and THF (24 h) and dried *in vacuo* to afford 442 mg (97%) of a dark blue powder.

2. Characterization of Synthesized Materials

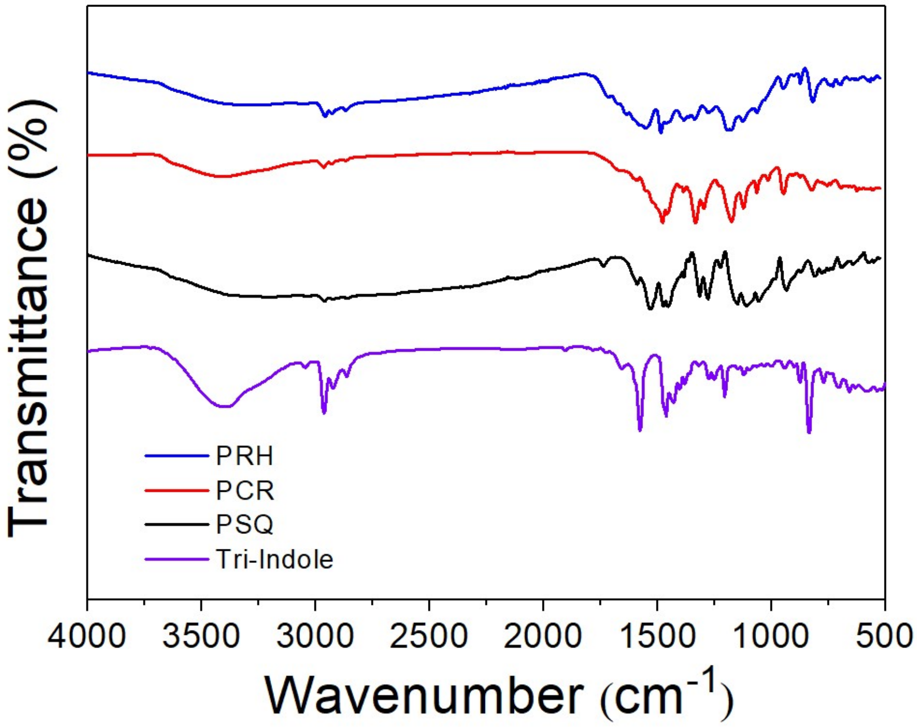

Fig. S2 FT-IR of the tri-substituted indole (1) and the three polymers: **PSQ**, **PCR**, and **PRH**.

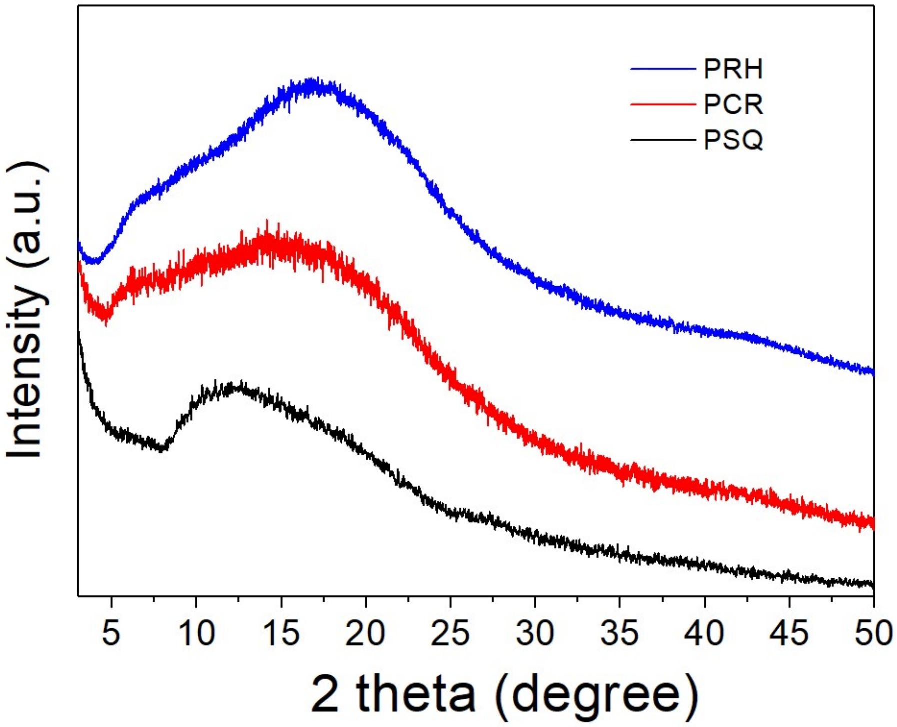

Fig. S3 PXRD patterns of **PSQ**, **PCR** and **PRH**.

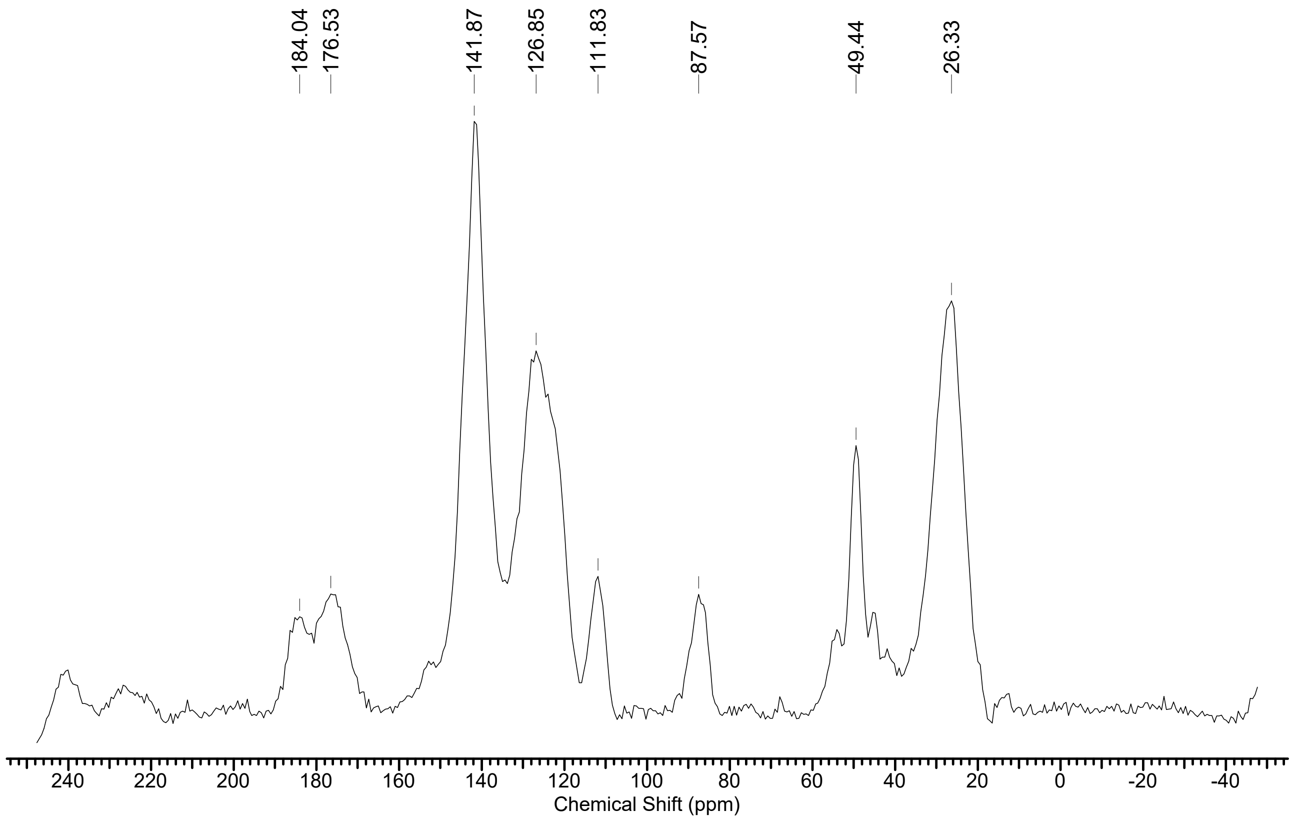

Fig. S4 Solid state ^{13}C NMR analysis of **PSQ**.

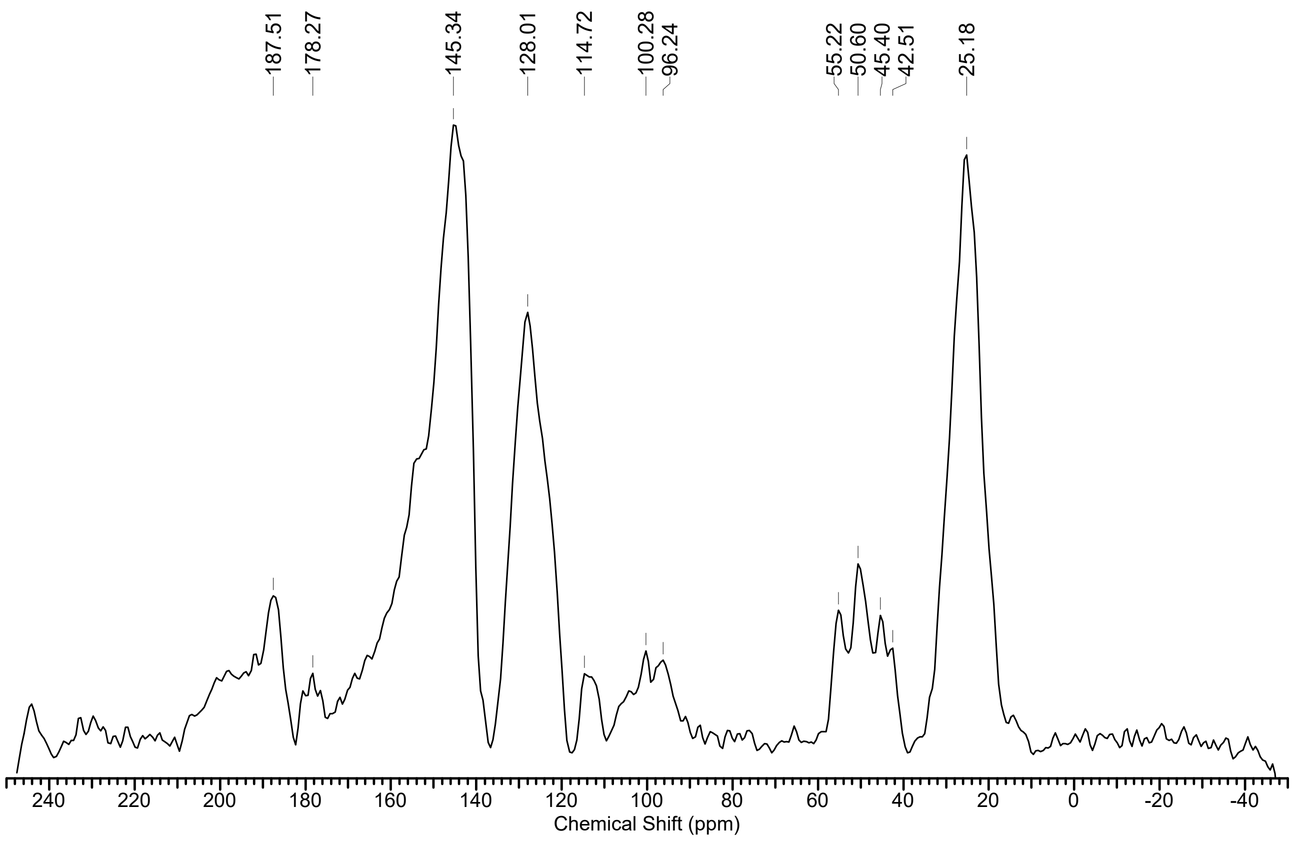

Fig. S5 Solid state ^{13}C NMR analysis of **PCR**.

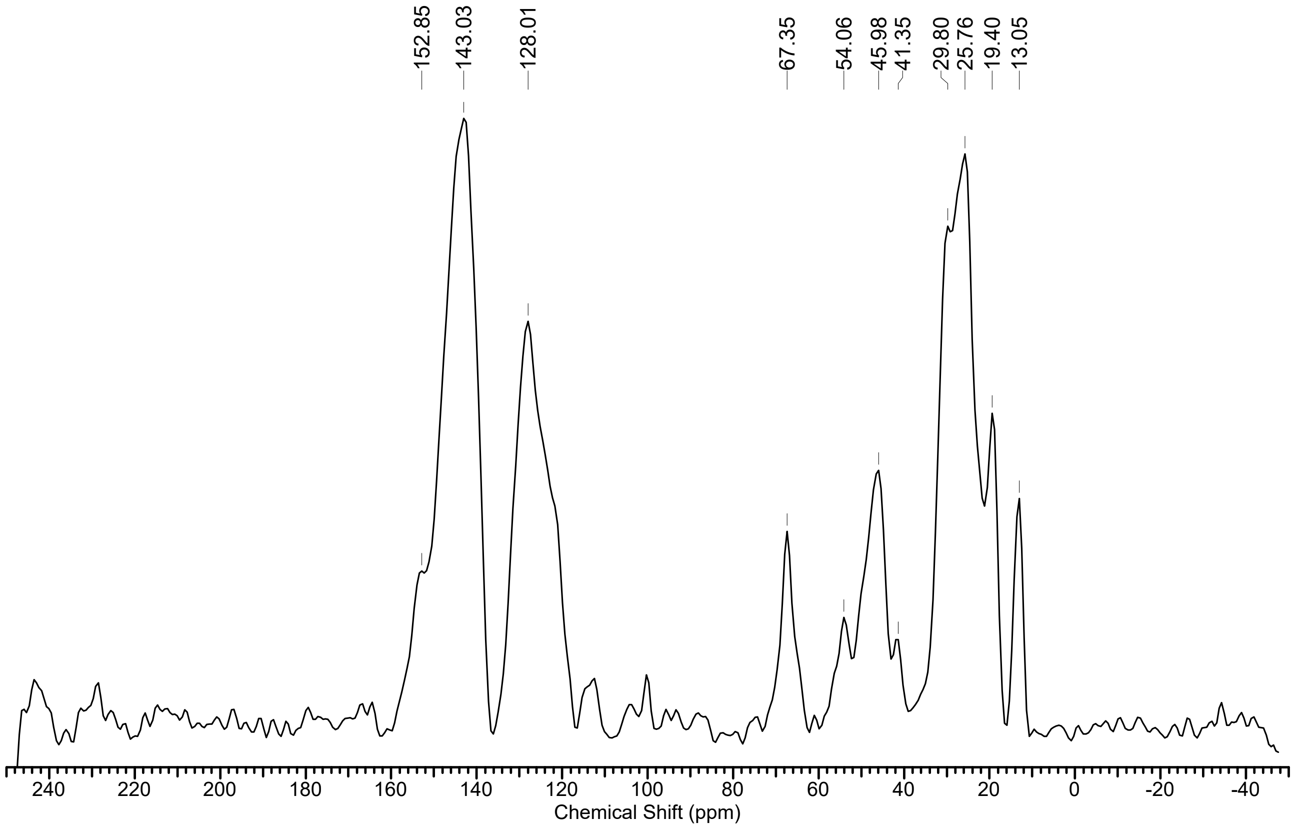

Fig. S6 Solid state ^{13}C NMR analysis of **PRH**.

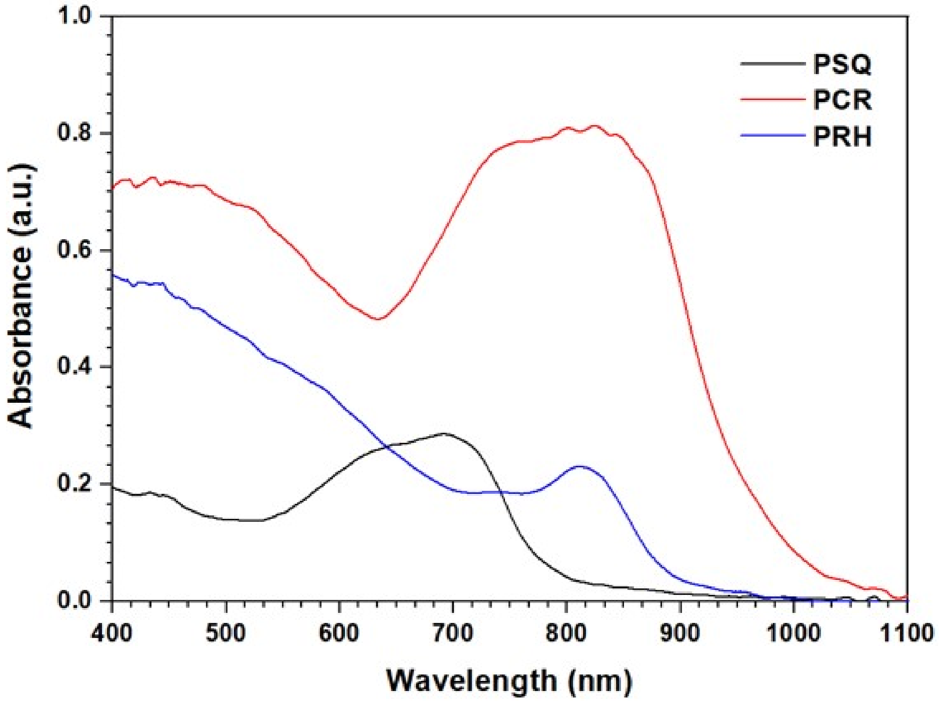

Fig. S7 UV-Vis spectra of **PSQ**, **PCR** and **PRH** with local λ_{max} ranging from 700-850 nm.

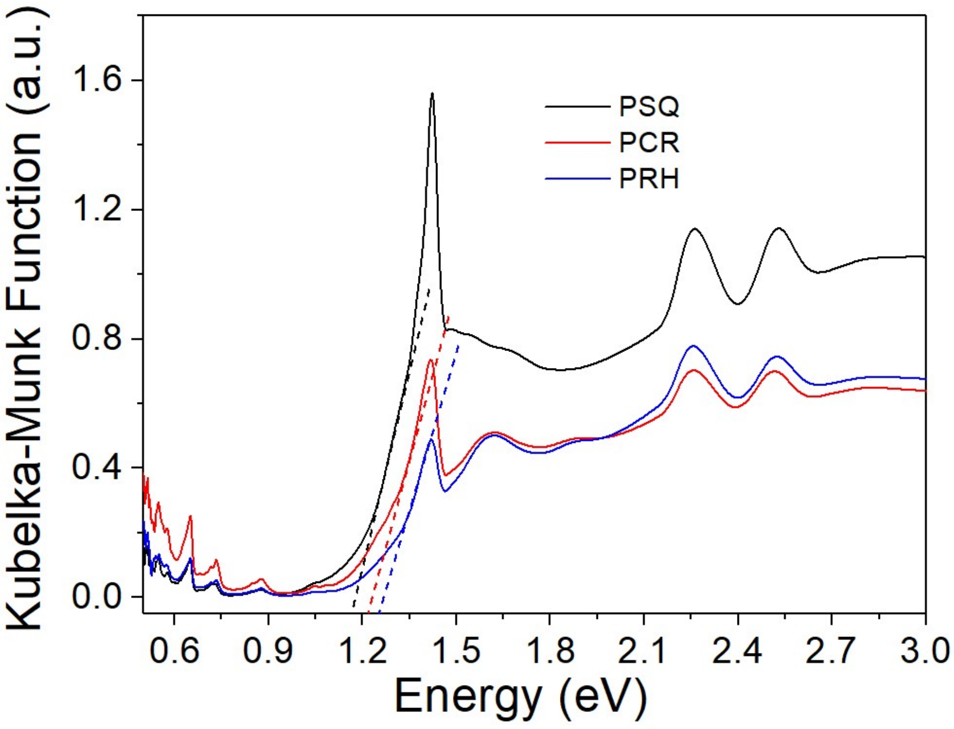

Fig. S8 Diffuse reflectance spectra for **PSQ**, **PCR** and **PRH**.

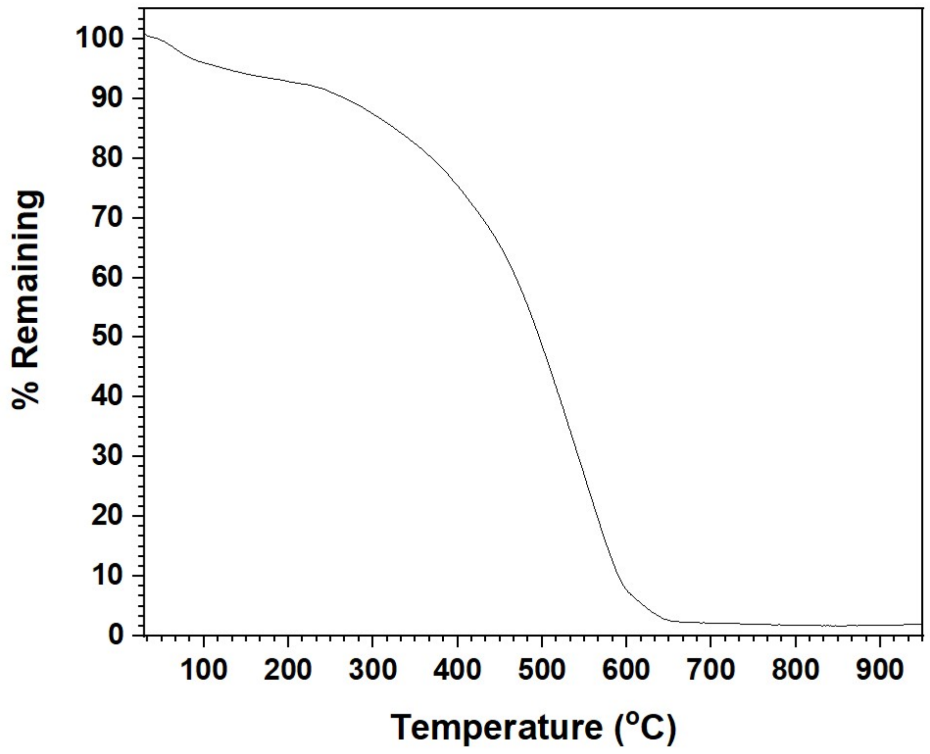

Fig. S9 TGA trace for **PSQ**.

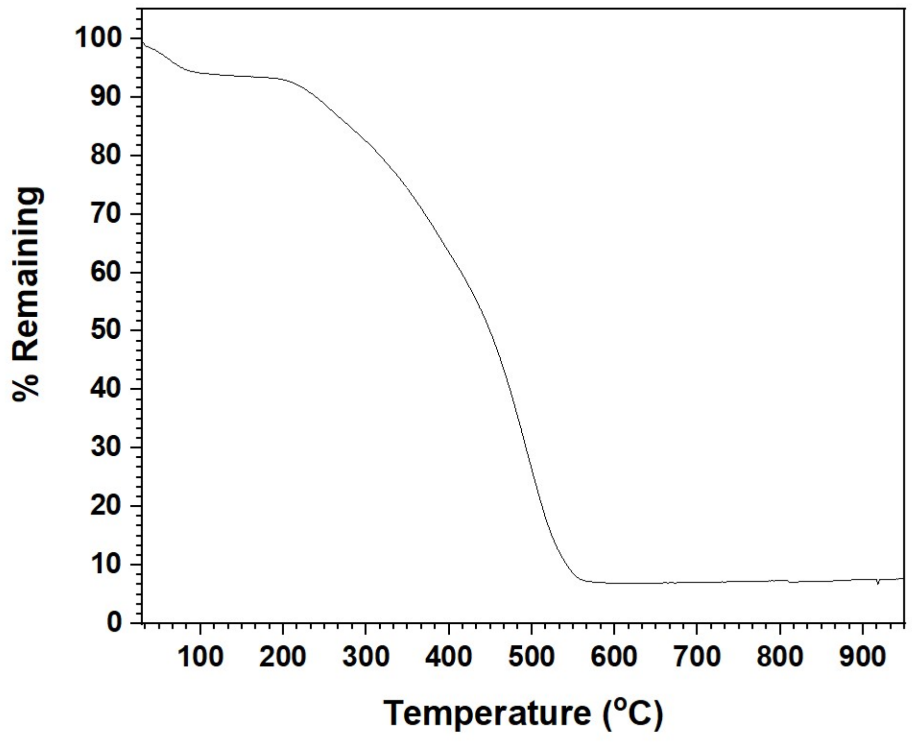

Fig. S10 TGA trace for **PCR**.

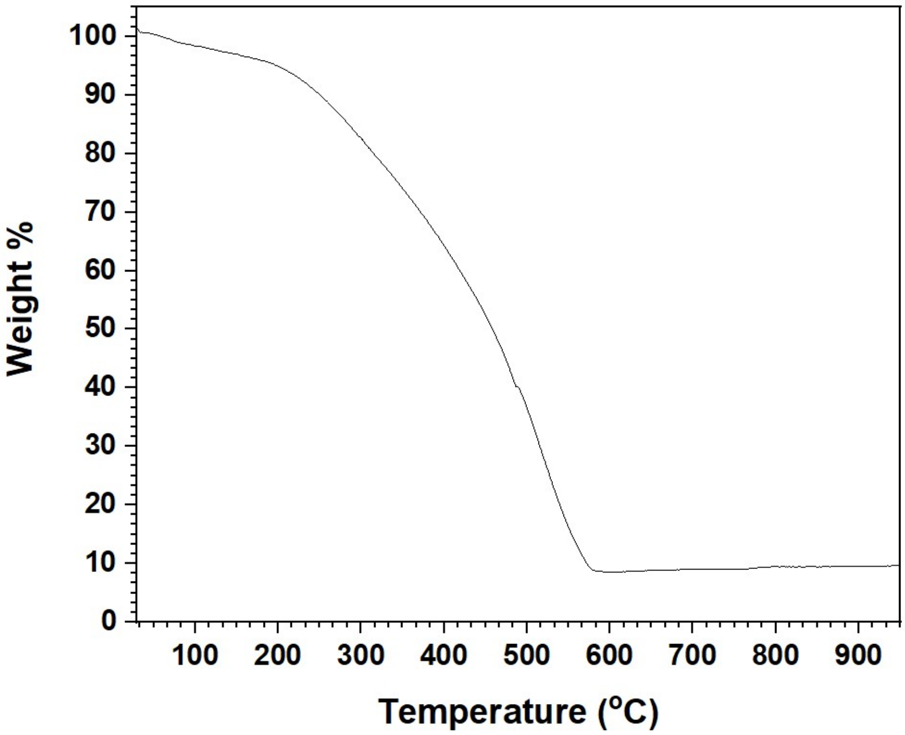

Fig. S11 TGA trace for **PRH**.

Procedures for the surface area analyses for PSQ, PCR and PRH. The porosity analyses were performed using a Quantachrome Autosorb iQ gas sorption analyzer. Each polymeric samples was outgassed at 0.03 torr with a 2 °C/min ramp to 100 °C and held at 100 °C for 12 hours. Pore analysis was first performed using CO_2 at 273.15 K. Afterwards, each sample was reactivated at 0.03 torr with a 2 °C/min ramp to 100 °C and held at 100 °C for 12 hours. Pore analysis was then performed using N_2 at 77.35 K (P/P_0 range of 1×10^{-5} to 0.995).

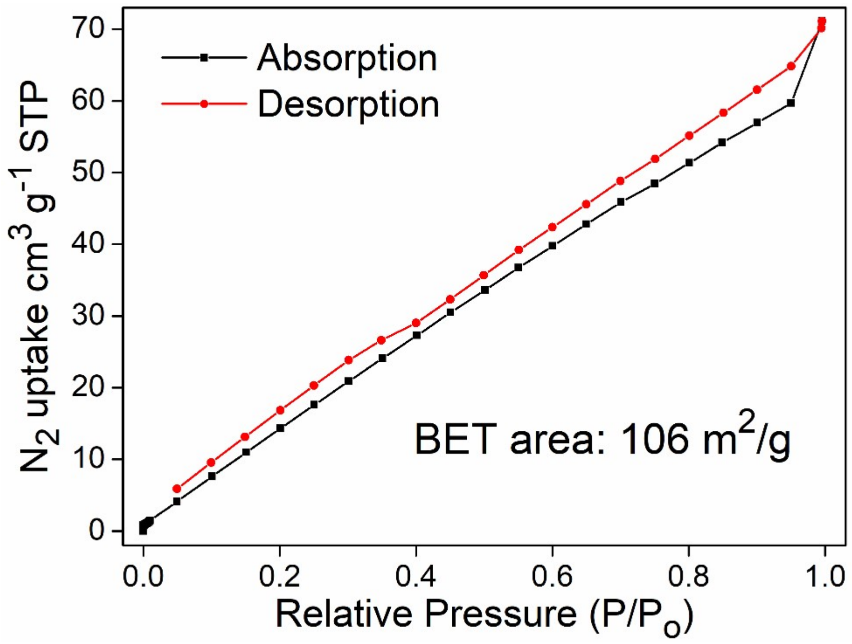

Fig. S12 N_2 sorption isotherms at 77 K for a sample of **PSQ** (80.2 mg). Inset: the corresponding Brunauer–Emmett–Teller (BET) surface area of 106 m^2/g.

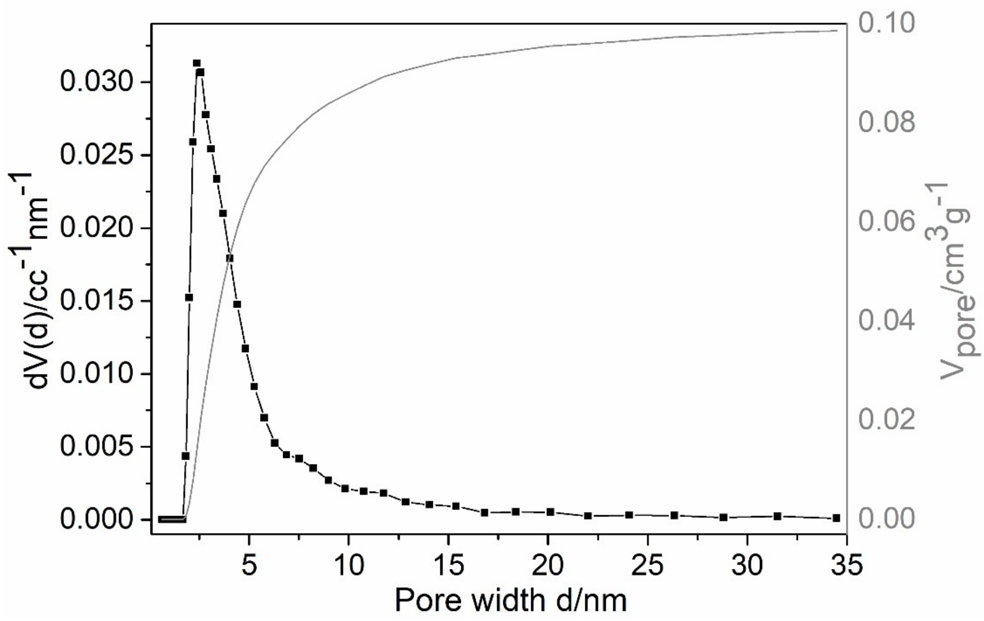

Fig. S13 Pore size distribution and pore volume of a sample of **PSQ** (N_2 gas at 77 K; QSDFT model). The average pore width 2.382 nm, the pore volume 0.099 cm^3/g.

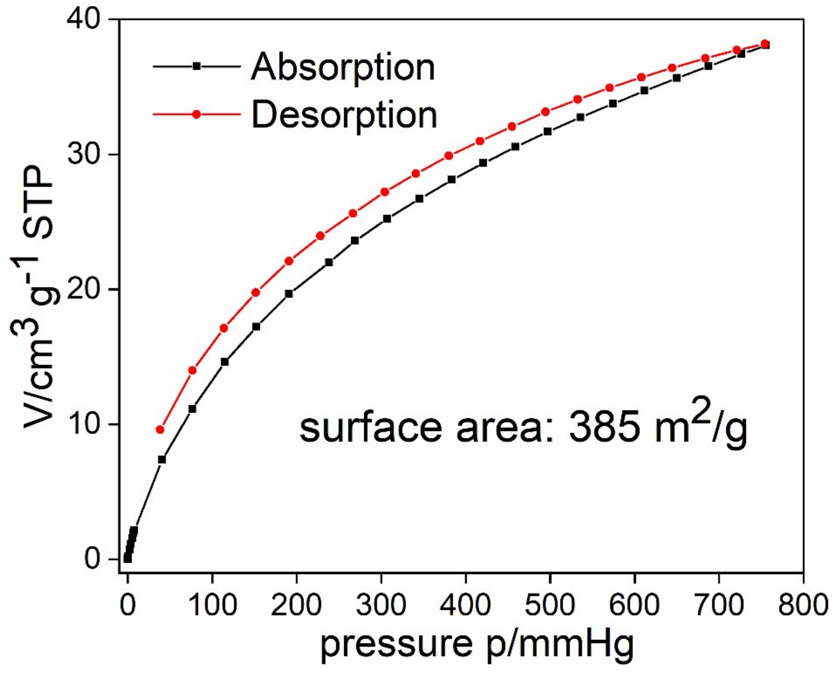

Fig. S14 CO_2 sorption isotherms at 273 K for a sample of **PSQ** (80.2 mg). Inset: the corresponding surface area of 385 m^2/g (calculated using Monte-Carlo method).

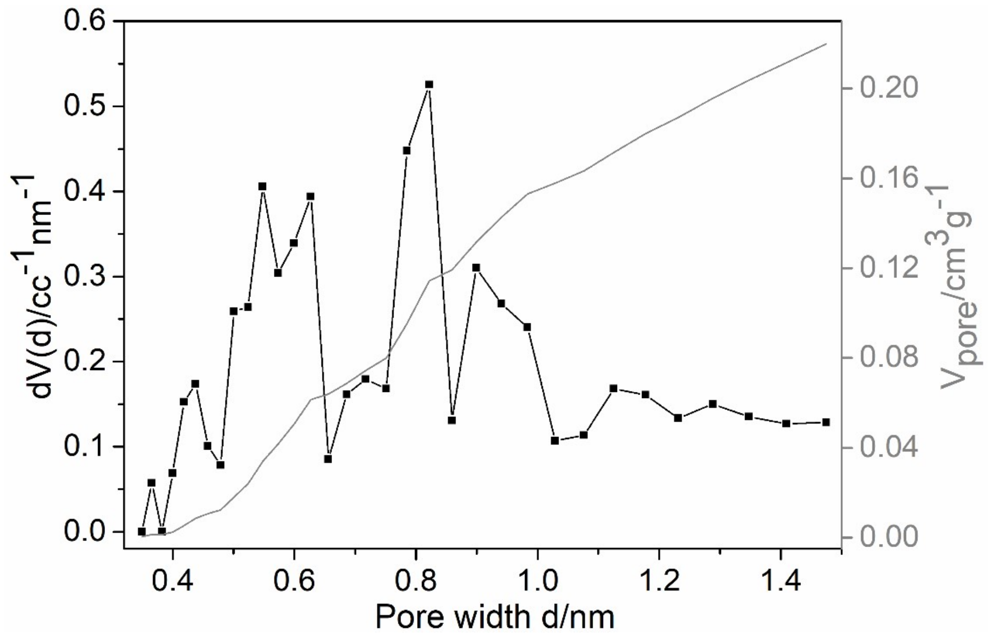

Fig. S15 Pore size distribution and pore volume of a sample of **PSQ** (CO_2 gas at 273 K; Monte-Carlo model). The average pore width 0.822 nm, the pore volume 0.220 cm^3/g.

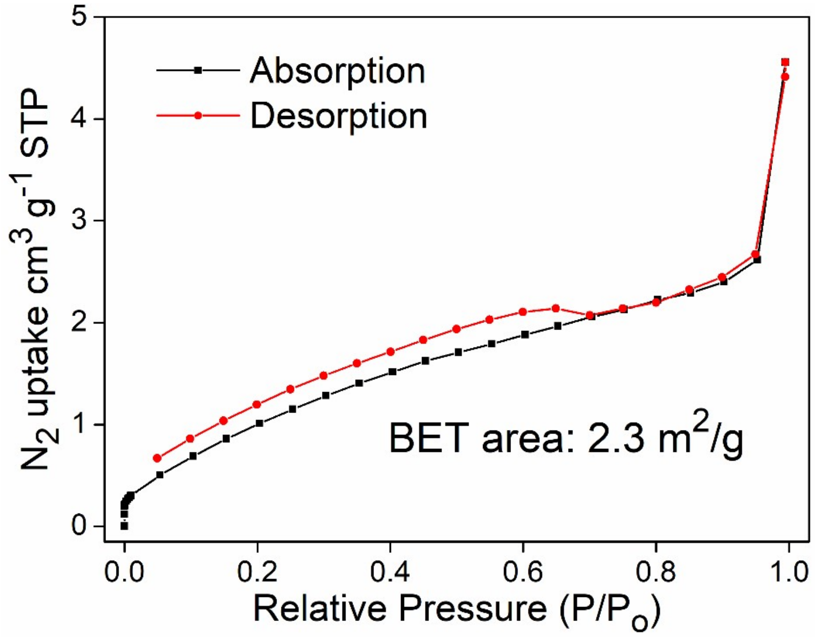

Fig. S16 N_2 sorption isotherms at 77 K for a sample of **PCR** (110.2 mg). Inset: the corresponding Brunauer–Emmett–Teller (BET) surface area of 2.3 m^2/g.

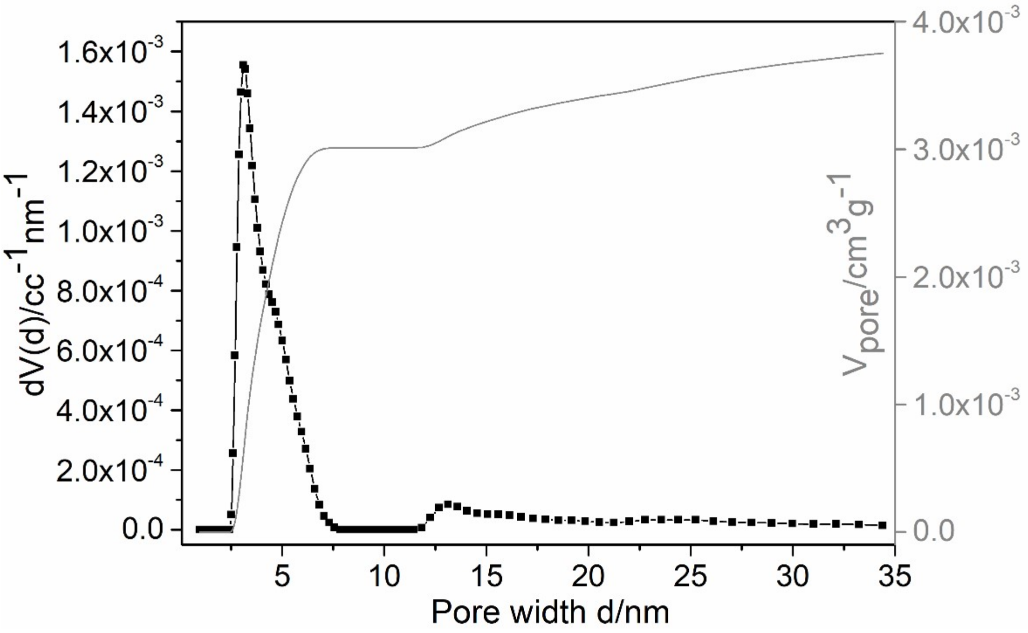

Fig. S17 Pore size distribution and pore volume of a sample of **PCR** (N_2 gas at 77 K; QSDFT model). The average pore width 3.096 nm, the pore volume 0.005 cm^3/g.

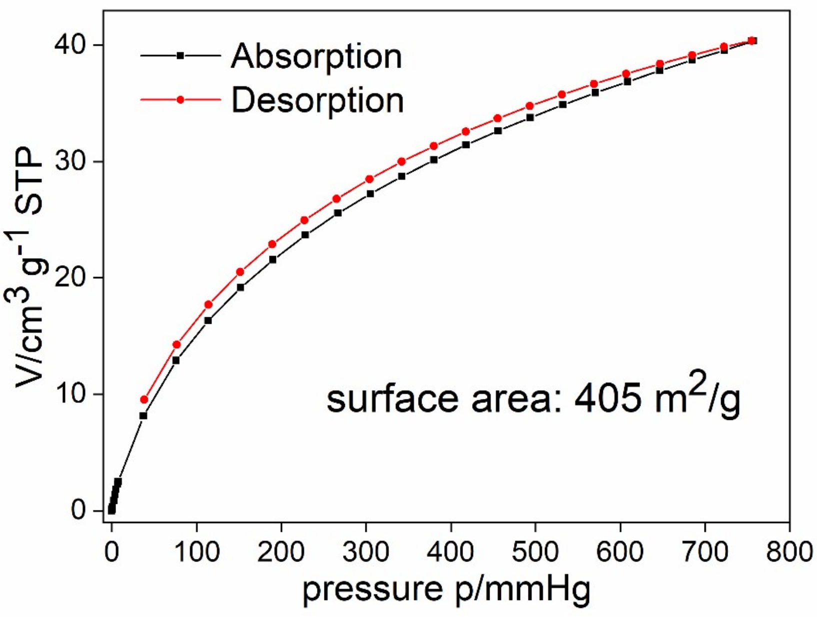

Fig. S18 CO_2 sorption isotherms at 273 K for a sample of **PCR** (110.2 mg). Inset: the corresponding surface area of 405 m^2/g (calculated using Monte-Carlo method).

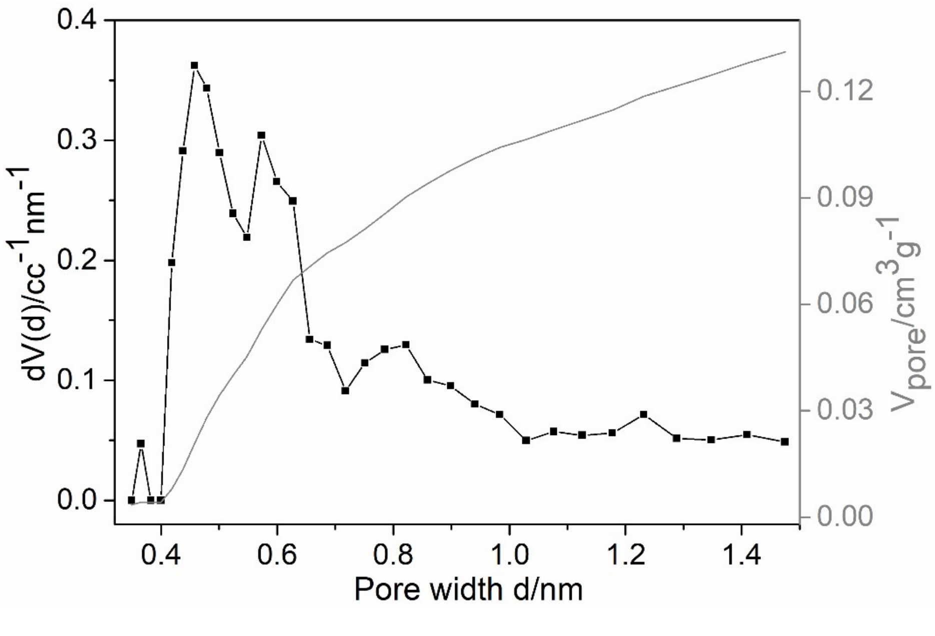

Fig. S19 Pore size distribution and pore volume of a sample of **PCR** (CO_2 gas at 273 K; Monte-Carlo model). The average pore width 0.458 nm, the pore volume 0.131 cm^3/g.

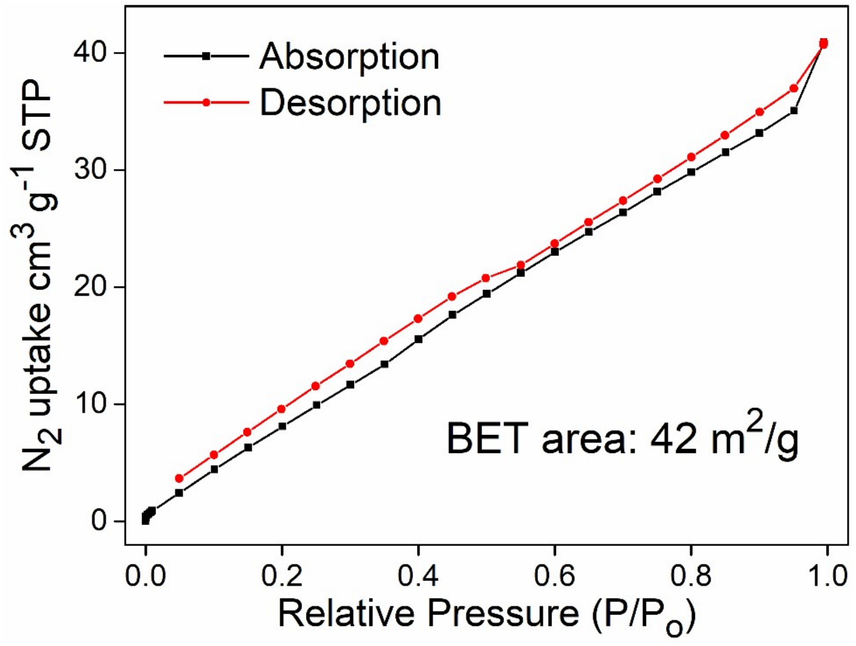

Fig. S20 N_2 sorption isotherms at 77 K for a sample of **PRH** (64.9 mg). Inset: the corresponding Brunauer–Emmett–Teller (BET) surface area of 42 m^2/g.

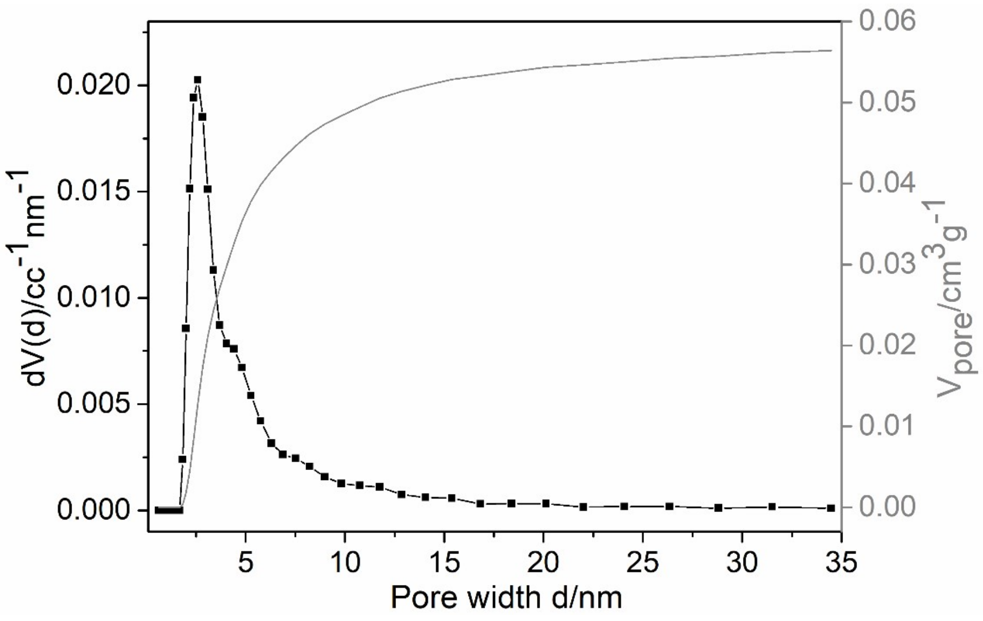

Fig. S21 Pore size distribution and pore volume of a sample of **PRH** (N_2 gas at 77 K; QSDFT model). The average pore width 2.6 nm, the pore volume 0.056 cm^3/g.

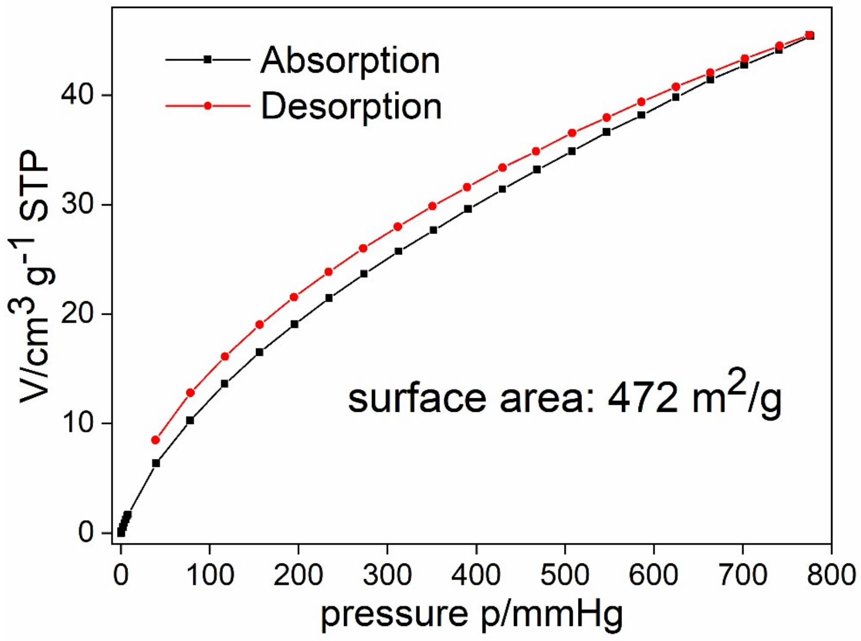

Fig. S22 CO_2 sorption isotherms at 273 K for a sample of **PRH** (58.9 mg). Inset: the corresponding surface area of 472 m^2/g (calculated using Monte-Carlo method).

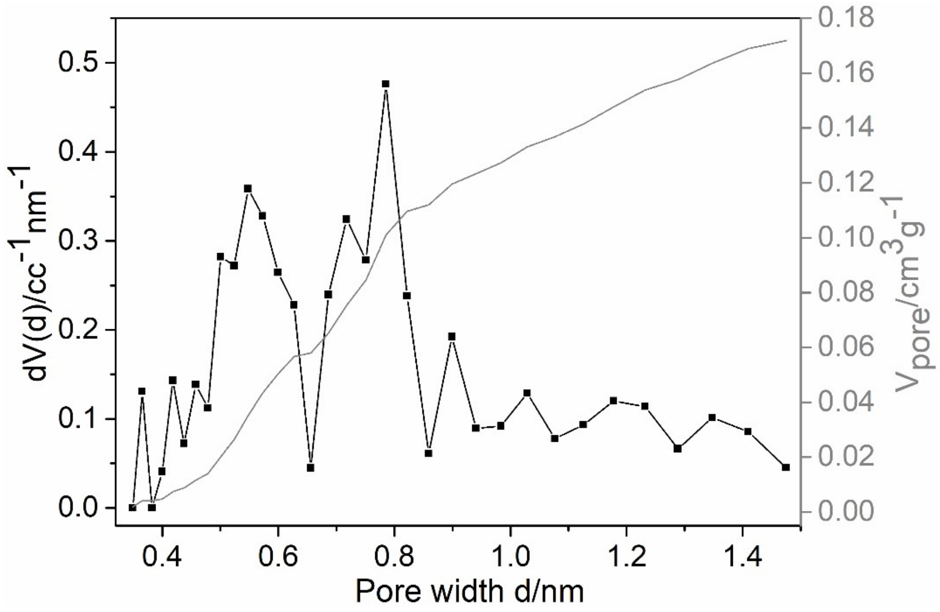

Fig. S23 Pore size distribution and pore volume of a sample of **PRH** (CO_2 gas at 273 K; Monte-Carlo model). The average pore width 0.785 nm, the pore volume 0.172 cm^3/g.

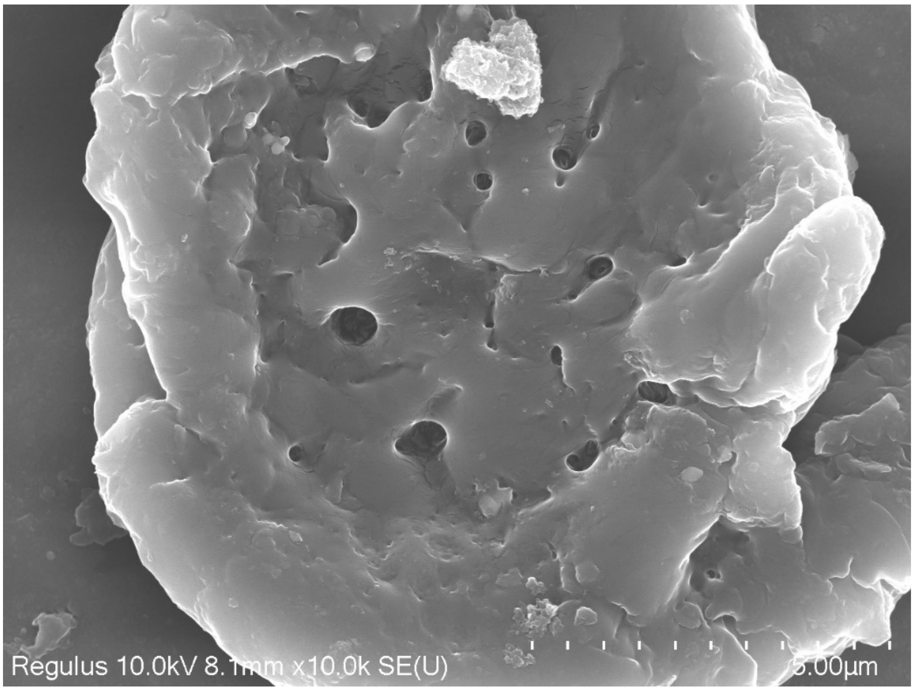

Fig. S24 SEM image of **PSQ**.

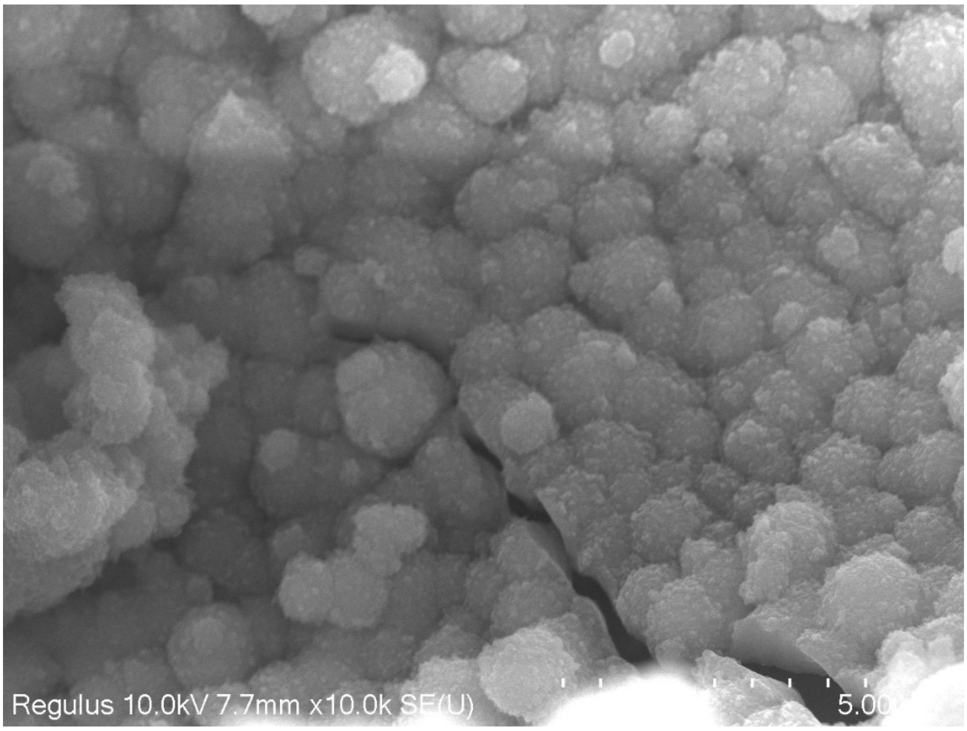

Fig. S25 SEM image of **PCR**.

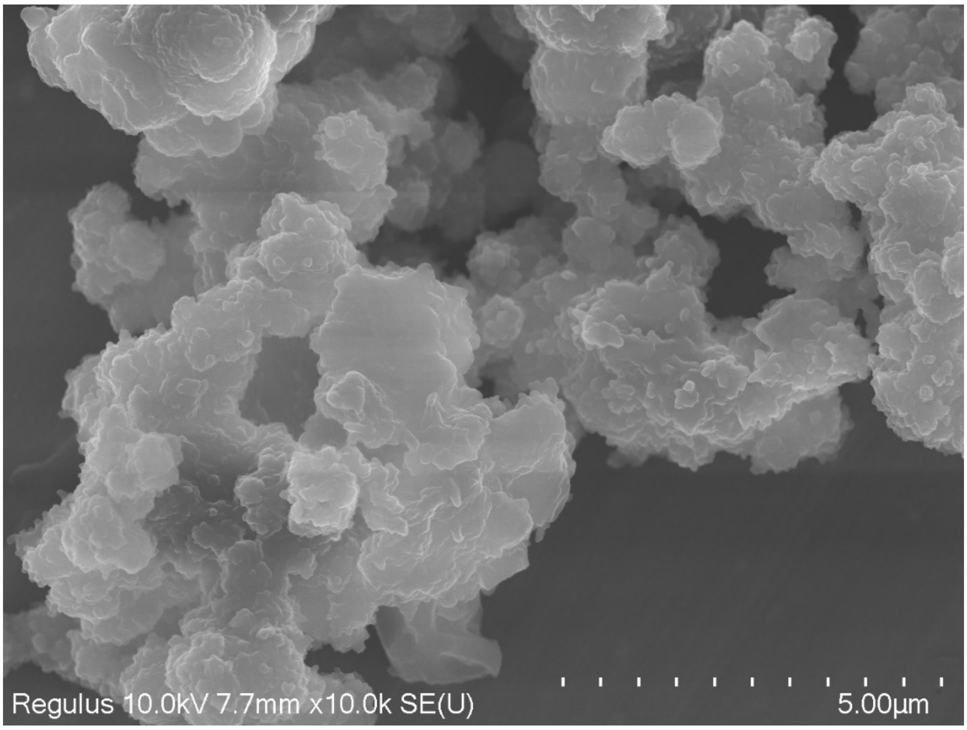

Fig. S26 SEM image of **PRH**.

3. Impedance Analysis

AC impedance data were obtained using a pellet (3 mm in diameter) pressed at 5,000 kg for a couple of minutes. The thickness of the pellet was ranging from 0.5 to 1.0 mm. The pellet was placed in a sample holder composed of electrode and the sample holder was put in a chamber controlling temperature and humidity. Data points were obtained after the condition to be stable over 30 min. Measurements were carried out using a Solartron SI 1260 Impedance/Gain-Phase Analyzer and 1296 Dielectric Interface and applied AC voltage amplitude of 100 mV and frequency range of 10 MHz - 1 Hz.

Synthesis of LiCl@PSQ

PSQ (15 mg) was ground for 30 min and then washed with deionised water (5 mL × 3) and acetone (5 mL × 3). After being dried in air, **PSQ** was immersed into saturated lithium chloride in THF (2 mL) for 2 days at room temperature. Then, the sample was filtered, washed with THF (5 mL × 3) and acetone (5 mL × 3), dried in air to afford the **LiCl@PSQ** sample.

Synthesis of LiCl@PCR

PCR (15 mg) was immersed into saturated lithium chloride in THF (2 mL) for 2 days at room temperature. Then, the sample was filtered, washed with THF (5 mL × 3) and acetone (5 mL × 3), dried in air to afford the **LiCl@PCR** sample.

Synthesis of LiCl@PRH

PRH (15 mg) was ground for 30 min and then washed with deionised water (5 mL × 3) and acetone (5 mL × 3). After being dried in air, **PRH** was immersed into saturated lithium chloride in THF (2 mL) for 2 days at room temperature. Then, the sample was filtered,

washed with THF (5 mL × 3) and acetone (5 mL × 3), dried in air to afford the **LiCl@PRH** sample.

Calculation for proton conductivity and activation energy.

The Nyquist plots (Z'' vs. Z') of proton-conducting MOF often show a single semicircle at high frequency, representing proton resistivity contributions of bulk sample. The proton conductivity was deduced from the semicircle by fitting an equivalent circuit which consists of Rs, R1 and W1 in the frequency range from 10 MHz to 1 Hz. Rs corresponds to wire and electrode resistance, R1 is proton resistance and W1 attributes to the resistivity of grain boundary. Sometimes W1 is not necessary, because the impedance plot of the capacitive tail may not appear in the measured range due to the high magnitude of the resistivity.

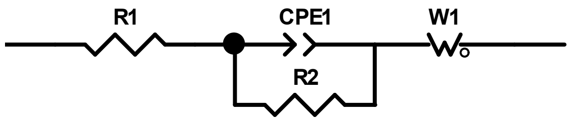

The water-assisted conductivities of synthesized materials were measured under different relative humidity and temperature conditions and were further fitted with different fitting circuits using the ZView software. Proton conductivity (σ, S cm^{-1}) was calculated from the impedance spectra with the equation of $\sigma = l/RS$, where l is the thickness (mm) and S is the cross-sectional area (mm^2) of the pellet, while R (Ω) can be calculated from the impedance plots. The activation energy values were calculated using the Arrhenius equation $\sigma T = \sigma_0 \exp(-Ea/kT)$ by the slope of the plots of ln(σT) versus 1000/T.

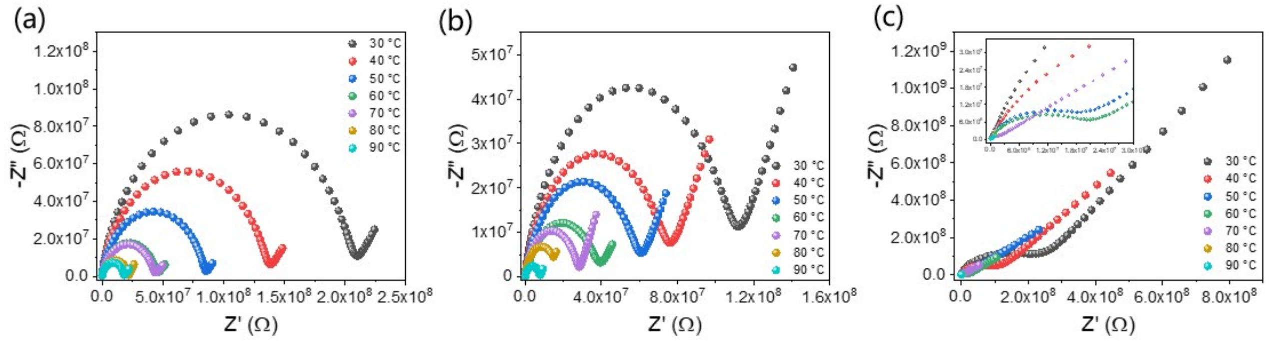

Fig. S27 Nyquist plots of (a) **PSQ**, (b) **PCR** and (c) **PRH** at different temperatures (from 30 °C to 90 °C) and 90% relative humidity.

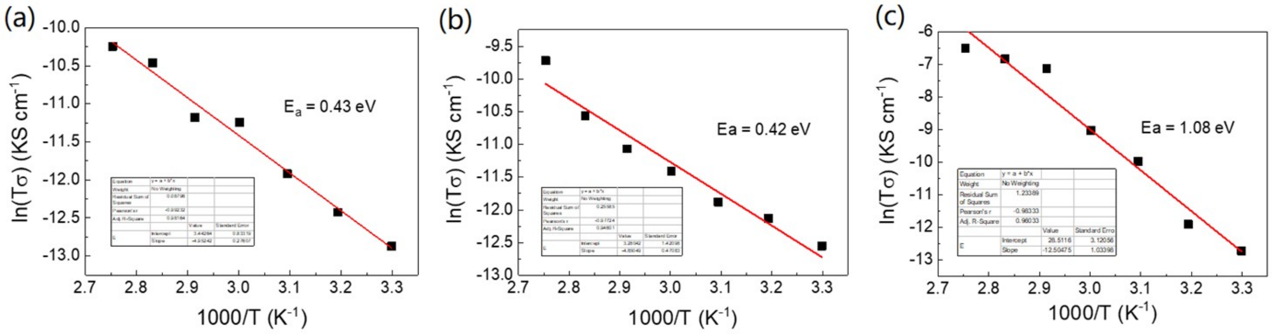

Fig. S28 Arrhenius plot of conductivities for bulk contribution to resistivity of (a) **PSQ**, (b) **PCR** and (c) **PRH** at 90% relative humidity.

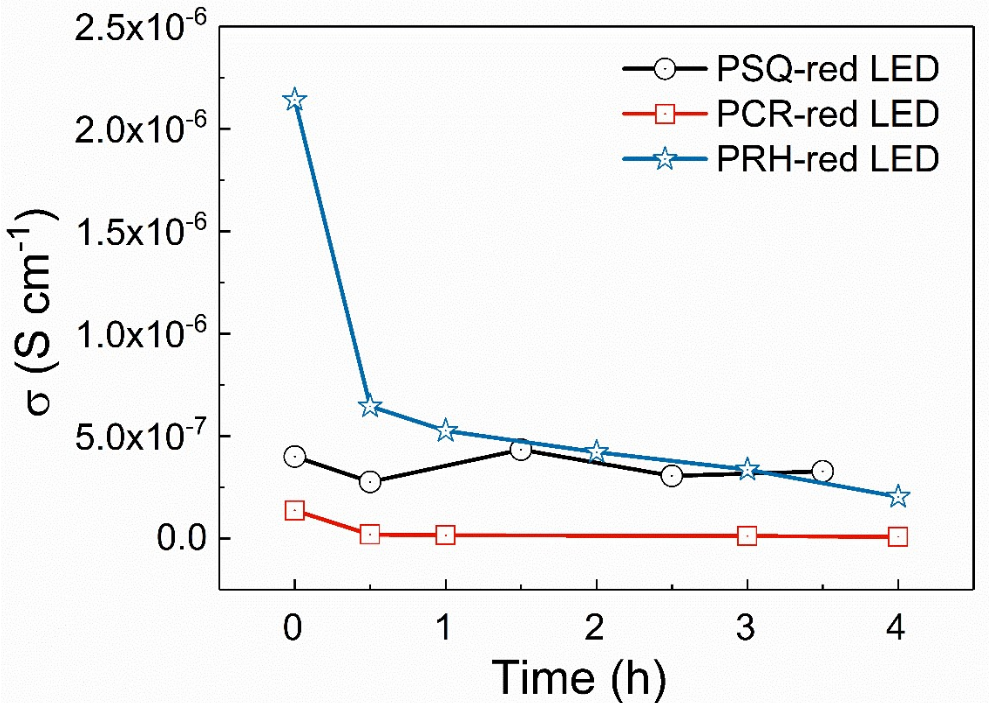

Fig. S29 Proton conductivity of **PSQ**, **PCR**, **PRH** after being illuminated by the red LED light for different time at 90 °C and 90% relative humidity.

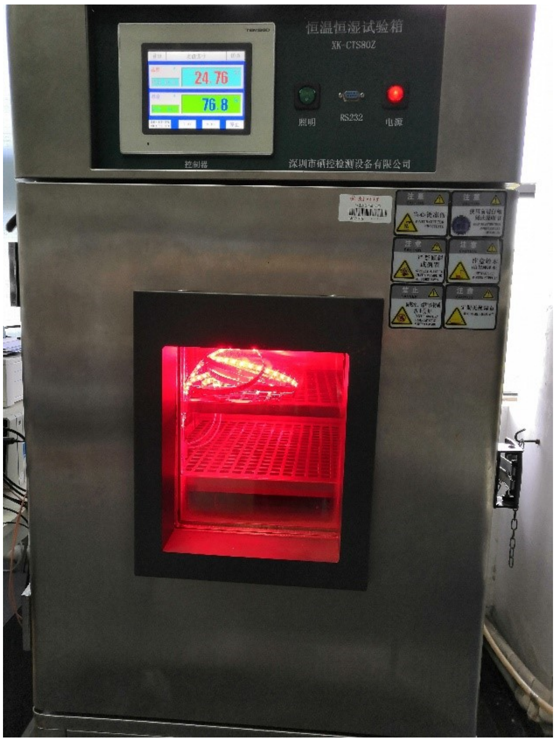

Fig. S30 A photograph of the device to measure impedance of sample after being illuminated by a red LED module.

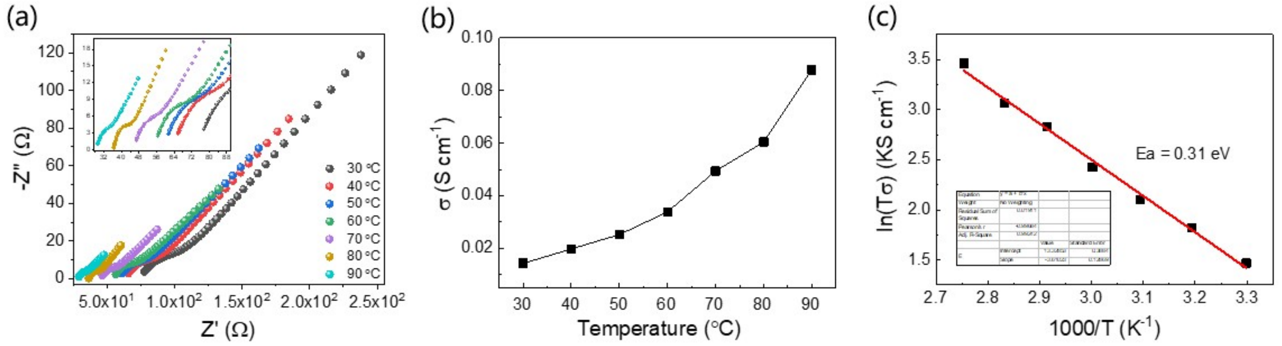

Fig. S31 (a) Nyquist plots and (b) proton conductivity of **LiCl@PSQ** at different temperatures (from 30˚C to 90˚C) and 90% relative humidity. (c) Arrhenius plot of conductivities for bulk contribution to resistivity of **LiCl@PSQ** at 90% relative humidity.

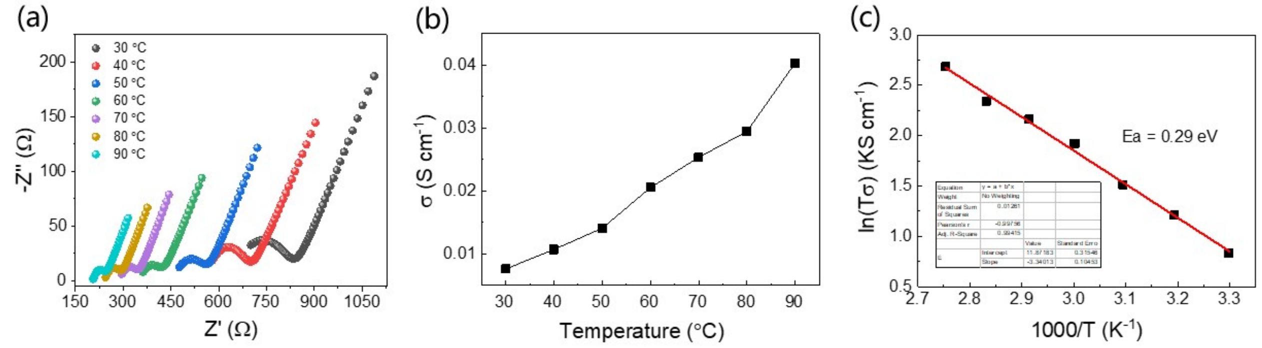

Fig. S32 (a) Nyquist plots and (b) proton conductivity of **LiCl@PSQ** at different temperatures (from 30˚C to 90˚C) and 40% relative humidity. (c) Arrhenius plot of conductivities for bulk contribution to resistivity of **LiCl@PSQ** at 40% relative humidity.

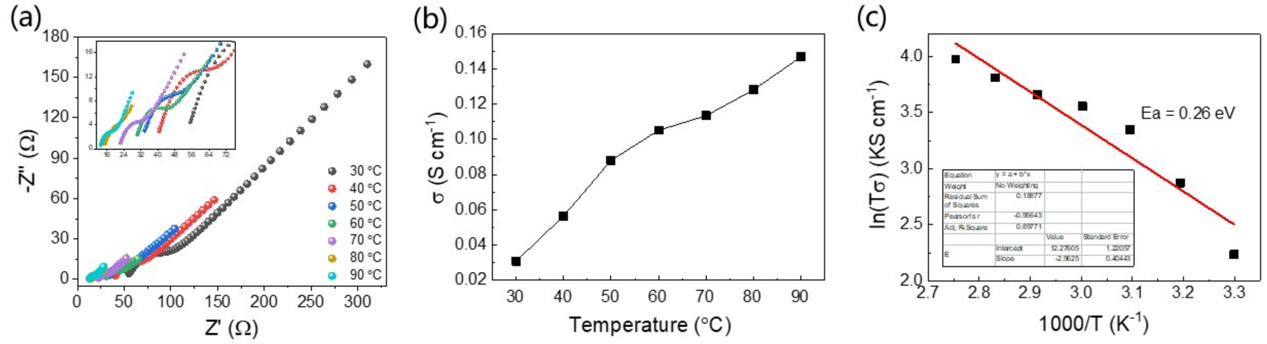

Fig. S33 (a) Nyquist plots and (b) proton conductivity of **LiCl@PCR** at different temperatures (from 30˚C to 90˚C) and 90% relative humidity. (c) Arrhenius plot of conductivities for bulk contribution to resistivity of **LiCl@PCR** at 90% relative humidity.

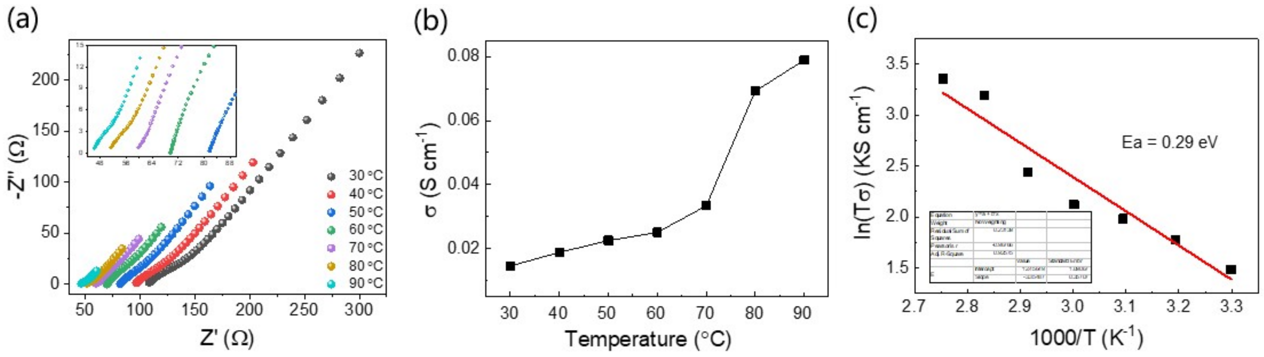

Fig. S34 (a) Nyquist plots and (b) proton conductivity of **LiCl@PCR** at different temperatures (from 30˚C to 90˚C) and 40% relative humidity. (c) Arrhenius plot of conductivities for bulk contribution to resistivity of **LiCl@PCR** at 40% relative humidity.

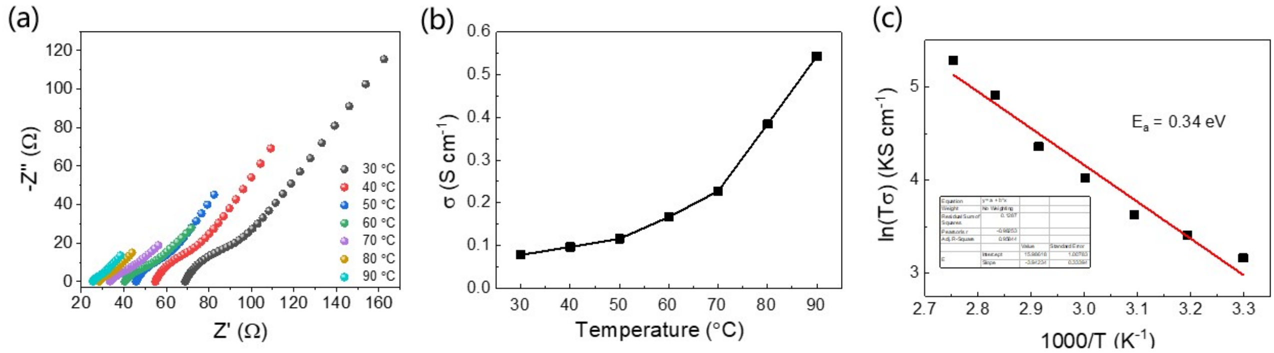

Fig. S35 (a) Nyquist plots and (b) proton conductivity of **LiCl@PRH** at different temperatures (from 30˚C to 90˚C) and 90% relative humidity. (c) Arrhenius plot of conductivities for bulk contribution to resistivity of LiCl@PRH at 90% relative humidity.

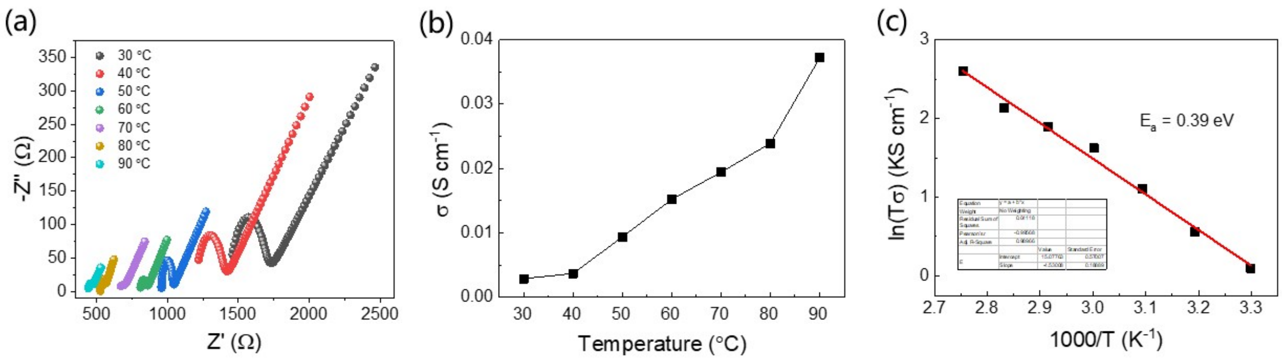

Fig. S36 (a) Nyquist plots and (b) proton conductivity of **LiCl@PRH** at different temperatures (from 30˚C to 90˚C) and 40% relative humidity. (c) Arrhenius plot of conductivities for bulk contribution to resistivity of LiCl@PRH at 40% relative humidity.

Table S1 Proton conduction results of **PSQ**, **PCR**, **PRH** and their corresponding samples after soaking in saturated LiCl in THF.

Sample	Soaking Medium	Soaking Temperature/ °C	Soaking Time	σ/ S cm^{-1}	Relative humidity/%	T/°C	E_a/eV
**PSQ**	-	-	-	9.8 x 10^{-8}	90	90	0.43
**LiCl@PSQ**	LiCl/THF	RT	2 days	0.088	90	90	0.31
**LiCl@PSQ**	LiCl/THF	RT	2 days	4.0 x 10^{-2}	40	90	0.29
**PCR**	-	-	-	1.9 x 10^{-7}	90	90	0.42
**LiCl@PCR**	LiCl/THF	RT	2 days	0.15	90	90	0.26
**LiCl@PCR**	LiCl/THF	RT	2 days	7.9 x 10^{-2}	40	90	0.29
**PRH**	-	-	-	4.1 x 10^{-6}	90	90	1.08
**LiCl@PRH**	LiCl/THF	RT	2 days	0.54	90	90	0.34
**LiCl@PRH**	LiCl/THF	RT	2 days	3.7 x 10^{-2}	40	90	0.39

Theoretically, the proton conductivity has a good correlation with the activation energy, i.e., the proton conductivities follow the order **PSQ** < **PCR** < **PRH**, the activation energy follow the order **PSQ** > **PCR** > **PRH** (*J. Am. Chem. Soc.* 2011, *133*, 2034–2036). However, the measured activation energies of **PSQ**, **PCR** and **PRH** at 90 °C were 0.43, 0.42 and 1.08 eV, respectively. As for the higher activation energy of **PRH**, one possibility is onset of a glass or phase transition that causes the sites preferred by protons in the polymer to become disordered. (*J. Am. Ceram. Soc.* 2002, *85*, 2637)

pH-Measurement of PSQ, PCR and PRH

10 mg of sample polymer was immersed into deionized water (0.2 mL). After sonication, the pH was measured using universal indicator paper (see Figure S37). Two key observations were drawn from this experiment: 1) The pH values all point to weak acidity (no lower than 4), indicating low concentrations of protons, and consistent with the low proton conductivities of all three as-made samples at room temperature. 2) The pH values of the **PRH** and **PCR** are slightly lower than that of **PSQ**, which could be caused by the greater number of electron-withdrawing C=O groups in the croconic (3 C=O groups) and rhodizonic (4 C=O groups) units, relative to the squaric (2 C=O groups) unit.

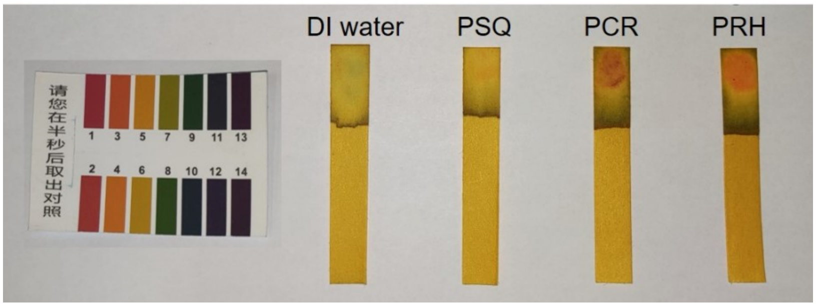

Fig. S37 pH Measurements of **PSQ**, **PCR** and **PRH**.
